# A novel HIF1α-STIL-FOXM1 axis regulates tumor metastasis

**DOI:** 10.1186/s12929-022-00807-0

**Published:** 2022-04-01

**Authors:** Yi-Wei Wang, Shu-Chuan Chen, De-Leung Gu, Yi-Chen Yeh, Jhih-Jie Tsai, Kuo-Tai Yang, Yuh-Shan Jou, Teh-Ying Chou, Tang K. Tang

**Affiliations:** 1 Institute of Biomedical Sciences, Academia Sinica, 128 Academia Rd., Sec. 2, Taipei, 11529 Taiwan; 2 Department of Pathology and Laboratory Medicine, Taipei Veterans General Hospital, Taipei, Taiwan; 3 Present Address: Dept. of Animal Science, National Pingtung University of Science and Technology, Pingtung, Taiwan

**Keywords:** Metastasis, STIL, FOXM1, HIF1α, Centrosome

## Abstract

**Background:**

Metastasis is the major cause of morbidity and mortality in cancer that involves in multiple steps including epithelial–mesenchymal transition (EMT) process. Centrosome is an organelle that functions as the major microtubule organizing center (MTOC), and centrosome abnormalities are commonly correlated with tumor aggressiveness. However, the conclusive mechanisms indicating specific centrosomal proteins participated in tumor progression and metastasis remain largely unknown.

**Methods:**

The expression levels of centriolar/centrosomal genes in various types of cancers were first examined by in silico analysis of the data derived from The Cancer Genome Atlas (TCGA), Gene Expression Omnibus (GEO), and European Bioinformatics Institute (EBI) datasets. The expression of STIL (SCL/TAL1-interrupting locus) protein in clinical specimens was further assessed by Immunohistochemistry (IHC) analysis and the oncogenic roles of STIL in tumorigenesis were analyzed using in vitro and in vivo assays, including cell migration, invasion, xenograft tumor formation, and metastasis assays. The transcriptome differences between low- and high-STIL expression cells were analyzed by RNA-seq to uncover candidate genes involved in oncogenic pathways. The quantitative polymerase chain reaction (qPCR) and reporter assays were performed to confirm the results. The chromatin immunoprecipitation (ChIP)-qPCR assay was applied to demonstrate the binding of transcriptional factors to the promoter.

**Results:**

The expression of *STIL* shows the most significant increase in lung and various other types of cancers, and is highly associated with patients’ survival rate. Depletion of STIL inhibits tumor growth and metastasis. Interestingly, excess STIL activates the EMT pathway, and subsequently enhances cancer cell migration and invasion. Importantly, we reveal an unexpected role of STIL in tumor metastasis. A subset of STIL translocate into nucleus and associate with FOXM1 (Forkhead box protein M1) to promote tumor metastasis and stemness via FOXM1-mediated downstream target genes. Furthermore, we demonstrate that hypoxia-inducible factor 1α (HIF1α) directly binds to the *STIL* promoter and upregulates STIL expression under hypoxic condition.

**Conclusions:**

Our findings indicate that STIL promotes tumor metastasis through the HIF1α-STIL-FOXM1 axis, and highlight the importance of STIL as a promising therapeutic target for lung cancer treatment.

**Supplementary Information:**

The online version contains supplementary material available at 10.1186/s12929-022-00807-0.

## Background

Cancer metastasis is a complex process consisting of many critical steps, such as migration, invasion, adhesion, and metastatic colonization [1]. Gain of migrating and invading abilities are the most important steps during the development of metastasis. Cells experiencing these alterations undergo profound morphological changes, collectively referred to as the epithelial–mesenchymal transition (EMT) process. EMT is considered to be a critical mechanism in regulating cancer invasion and metastasis [1, 2]. It can be triggered by oncogenic activation or microenvironmental stimuli, such as hypoxia. Indeed, the hypoxia of the tumor microenvironment has been shown to be closely associated with metastasis [3]. Under hypoxic conditions, hypoxia-induced factor-1α (HIF-1α) becomes stabilized and up-regulates a number of EMT-related transcription factors (e.g., TWIST and SNAIL) that promote tumor metastasis [4, 5].

Cancer stem cells are a small population of cancer cells holding stemness properties known as cancer stemness (CS), which possess the ability to self-renew and contribute to unlimited cancer proliferation, tumor aggressiveness, drug treatment resistance, and metastasis [6, 7]. Cancer stem cells have been demonstrated to be regulated by several pluripotent transcription factors, such as OCT4, SOX2, and NANOG [8, 9]. FOXM1, a member of the Forkhead box (FOX) family of transcription factors, was reported to be transcriptionally regulated by HIF1α under hypoxic conditions [10, 11] and overexpressed in many types of cancers including liver cancer, breast cancer, and lung cancer [12–14]. Upregulation or activation of FOXM1 in cancer plays a key role in numerous phenotypes including cell proliferation, CS, invasion, metastasis, and angiogenesis [15–18].

The centrosome, which is the major microtubule (MT)-organizing center, is composed of two centrioles surrounded by pericentriolar material (PCM) and plays important roles in cell division, polarity, and motility. The centrioles are highly conserved MT-based organelles that form the core of the animal centrosome and serve as templates for the formation of cilia and flagella [19]. Centrosome/centriole abnormality has been considered a major contributing factor that causes aneuploidy, cancers, primary microcephaly, and ciliopathies [20–22]. Previous elegant works have identified at least four functionally conserved core proteins (Zyg-1/SAK/PLK4, Sas-4/DSas-4/CPAP, Sas-5/Ana2/STIL, and Sas-6/DSas-6/SAS6) that are essential for centriole duplication in worm, fly, and human [23–25]. Among these, PLK4 (Polo-like kinase 4), CPAP (Centrosomal protein 4.1-associated protein), STIL, and SAS6 (Spindle assembly abnormal protein 6) are the human homologs. PLK4, a member of the Polo-like kinase family, is a master regulator of centriole biogenesis. In human cells, PLK4 is recruited to the proximal end of the mother centriole in G1 phase by CEP152 (Centrosomal protein 152) and CEP192 (Centrosomal protein 192) [26–29]. The onset of centriole assembly is triggered by the STIL-mediated PLK4 activation at the base of mother centriole [25] followed by the recruitment of SAS6, which in turn initiates the assembly of cartwheel, a structural platform for the procentriole formation [25]. CEP135 could then facilitate the stabilization of the procentriole’s cartwheel by interacting with SAS6, CPAP, and microtubules [30]. Once the cartwheel has assembled, CPAP then cooperates with CEP120 (Centrosomal Protein 120) and SPICE1 (Spindle and centriole-associated protein 1) to promote the assembly of centriolar microtubules in a newborn (daughter) centriole during S/G2 phase [31, 32]. Furthermore, STIL is also essential for the recruitment of RTTN (Rotatin) to the newborn centrioles [33], where the latter is required for the proper loading of CEP295 (Centrosomal protein 295) [34] to the proximal end centriole and two later-born centriolar proteins [e.g., POC5 (Proteome of centriole protein 5), POC1B (Proteome of centriole protein 1 beta), and CETN1 (Centrin 1)] to the distal-half centrioles at later stages [33]. Interestingly, inactivating mutations in *PLK4, STIL, CPAP* (also known as *CENPJ*), or *SAS6* can lead to autosomal recessive primary microcephaly (MCPH) in humans [35], while their overexpression can induce centriole amplification, centriole elongation, and/or centrosome aberrations usually accompanied by structural and/or numerical abnormalities [20, 22]. Centriole biogenesis must be tightly regulated to ensure that each centriole duplicates only once per cell cycle. However, supernumerary centrosomes are frequently observed in human tumors, and have been correlated with advanced tumor grade and poor prognosis [20, 36]. PLK4, which is a known key initiator of centriole biogenesis, is up-regulated in many different types of cancers, and PLK4 overexpression-induced centrosome amplification seems to be sufficient to drive tumorigenesis in mice in both p53-wild type [37] and p53-null background [38, 39].

STIL also known as SIL, was originally identified at the site of a genomic rearrangement in a T-cell acute lymphoblastic leukemia patient [40], which has been implicated in regulating centrosome integrity and mitotic spindle organization [41]. We and others previously showed that excess STIL induces centriole overamplification [42–44] and that STIL plays a key role in assembling a full-length centriole via interacting with two other microcephaly proteins, CPAP [43] and RTTN [33]. Later studies further demonstrated that STIL is a master controller that regulates PLK4 kinase activity [45–49] to initiate centriole duplication. Interestingly, STIL upregulation has been found in many types of cancers [50, 51]. However, little is known about its role in tumorigenesis.

Centrosome amplification is considered to be a hallmark of cancer. It is interesting that STIL loss-of-function causes MCPH, whereas abnormally high expression of STIL triggers centriole amplification [42–44] and is frequently observed in human cancers [50, 51]. In this study, we screened a number of centriolar/centrosomal genes involved in centriole biogenesis and examined their possible association with oncogenic transformation. Our results showed that STIL was up-regulated in multiple types of cancers, displaying the most significant increase in lung cancer patients. Importantly, we discovered a novel and unexpected role of STIL in tumor metastasis. We found that cells exposure to hypoxia or overexpression of HIF1α lead to the increased STIL expression, suggesting that STIL is up-regulated by HIF1α under hypoxia. We further demonstrated that a subset of STIL translocate into nucleus and associate with FOXM1 to promote tumor metastasis and CS via FOXM1-mediated downstream target genes.

## Methods

### Bioinformatics analysis

The microarray data of 163 paired lung cancer patients and their adjacent non-malignant lung tissues were derived from 4 GEO datasets: GSE33356 [52–54], GSE27262 [55, 56], GSE18842 [57], and GSE51024 [58]. These datasets were designed for the independent research purpose, which shared with only one pathological feature (paired NSCLC samples). The data of *STIL* expression analyzed by the microarray method of 9 normal lung cell lines and 166 lung cancer cell lines were derived from GEO dataset: GSE36133 [59]. After the microarray data was downloaded from GEO, the intensity was normalized by dChip software and adjusted by median probe. The RNA-seq data for genes expression in various types of cancers (Additional file 1: Table S1a) were obtained from TCGA dataset (https://www.cancer.gov/about-nci/organization/ccg/research/structural-genomics/tcga). To examine the *STIL* expression in primary lung cancers and distant metastases, the microarray data of 955 lung adenocarcinomas (ADCs) (GSE43580 [60], GSE30219 [61], GSE31546, GSE68465 [62], GSE37745 [63–66], GSE50081 [67], GSE27716 [68], GSE33532, GSE27716 [68] and GSE14814 [69]), and 28 brain metastases from lung ADCs (GSE14108) [70] were derived from GEO. These datasets were designed for the independent research purpose, which shared with only one pathological feature (lung ADCs). The data of Kaplan–Meier analysis of overall survival (Additional file 1: Table S1b and Additional file 2: Fig. S1) were derived from Kaplan–Meier Plotter dataset that is capable to assess the correlation between the expression of genes and survival in cancer patients (https://kmplot.com/analysis/index.php?p=background) [71]. Gene expression was categorized into high and low groups according to the ‘Auto select best cutoff’ value. To search for the STIL-correlating genes, the microarray data of lung cancer cell lines were derived from EBI: E-MTAB-37 [72]. To analyze the *STIL* DNA copy numbers, the SNP array data of lung cancer cell lines were derived from EBI (E-MTAB-38), and the data of primary lung cancers were derived from GEO (GSE62407 [73], GSE77684, GSE20584 [74], GSE20585 [74], GSE25016 [75], GSE33355 [52–54], GSE33848 [76], GSE36363 [77], and GSE72192 [78]). To dissect the DNA methylation status of *STIL*, the data of 450K methylation array in lung cancer cell lines (GSE36216 [79]), normal lung tissues (GSE52401 [80] and GSE56044 [81]), and lung cancer tissues (GSE56044 [81]) were derived from GEO. The histologic subtypes of lung cancer are described in Additional file 1: Table S2.

### Human specimens

Lung cancer tissue arrays (Luc1021, Luc1501, and Lum 961) were purchased from Pantomics and LCN241, LC814a, and LC817b were purchased from US Biomax. These arrays included 41 normal lungs, 280 lung cancers, and 115 matched metastatic lymph nodes.

### Immunohistochemistry (IHC)

An IHC assay was used to examine the protein expression levels of interest in human lung cancer arrays. After deparaffinization, the sections were treated with 3% H_2_O_2_ solution to inactivate the endogenous peroxidase activities, and then incubated in 0.01 M sodium citrate buffer for antigen retrieval. The sections were incubated with the indicated primary antibodies diluted by the primary antibody diluent (SCY Tek; Cat# ATG125) at 4 °C. After overnight reaction, the sections were further incubated with the secondary antibody and stained using the REAL EnVision Detection System (Dako; Cat# K5007) and Mayer’s hematoxylin solution (Sigma; Cat# MHS16). The immunohistochemical stained slides were digitized using a slide scanner (Leica; Aperio AT2), and the digital slide images were assessed by the pathologist, without knowledge of other clinical data. The staining of the tumor cells was scored on a categorical scale: 0 (no staining), + (weak staining), ++ (moderate staining), or +++ (strong staining), as previously described [82].

### Cell culture and transfection

The human lung cancer cell lines, NCI-H1299, NCI-H2009, CL1-0, CL1-3, and CL1-5, were maintained in RPMI supplemented with 10% fetal bovine serum (FBS) and penicillin–streptomycin at 37 °C. HEK293T cells were maintained in Dulbecco’s Modified Eagle’s Medium (DMEM) supplemented with 10% fetal bovine serum and penicillin–streptomycin at 37 °C. The NCI-H1299, NCI-H2009, and HEK293T cells, originally obtained from ATCC, were previously described [83]. The CL1-0, CL1-3, and CL1-5 cells were kindly provided by Dr. Pan-Chyr Yang [84]. The cells were cultured under the normoxic (20% O_2_) or hypoxic (1% O_2_) conditions as indicated. Cells were transiently transfected with various cDNA constructs using Lipofectamine 2000 (Invitrogen; Cat# 11668). Cell lines were authenticated with short tandem repeat markers according to ATCC information and tested negative for mycoplasma contamination.

### Antibodies and constructs

The antibodies used in this study are listed in Additional file 1: Table S3. For generation of inducible GFP-STIL cell lines, the pLVX-Tight-Puro/STIL plasmid was generated by insertion of the full-length *STIL* cDNA into the pLVX-Tight-Puro vector (BD Bioscience). For the *STIL* promoter assay, the pGL3-*STIL* promoter-luciferase plasmid was constructed by insertion of PCR amplification of *STIL* promoter region into pGL3-basic vector (Promega). Four mutations of HIF1α DNA-binding sites in the *STIL* promoter (site1: nts − 195 to − 199; site2: − 894 to − 898; site3: − 1054 to − 1058; and site4: − 1235 to − 1239) were generated from pGL3-*STIL* wild-type promoter-luciferase construct using the QuikChange Lightning Site-Directed Mutagenesis Kit (Agilent; Cat# 210519). The cDNA constructs for various Flag-tagged STIL truncated mutants were described previously [43]. The pHA-HIF1α (ΔODD) construct was a gift from Dr. Muh-Hwa Yang [5]. The pGL3-*SLUG* promoter-luciferase construct was kindly provided by Dr. Cheng-Wen Wu [85]. The promoter-luciferase constructs of *NANOG*, *SOX2*, and *POU5F1* were previously described [86]. For in vivo metastasis assay, the pEIF4G-As-Luc2 plasmid was a gift from Dr. Ruey-Hwa Chen [87].

### Lentivirus production and infection

For generation of recombinant lentivirus, HEK293T cells were co-transfected with the packaging plasmids, pMD.G and pCMVΔR8.91 (provided by National RNAi Core Facility at Academia Sinica), and the indicated expression constructs, using Lipofectamine 2000 (Invitrogen; Cat# 11668). Two days after transfection, the virus-containing media were harvested and filtered. For lentivirus infection, the indicated cells were infected with lentivirus-containing media in the presence of 8 µg/mL polybrene.

### Generation of doxycycline (Dox)-inducible GFP-STIL cell lines

To obtain the CL1-0 and NCI-H1299 cell lines inducibly expressing GFP-STIL under Dox stimulation (Clontech; Cat# 8634-1), the pLVX-Tet-On (BD Biosciences) plasmid was introduced into the desired cells by lentivirus infection to establish the Tet-On cells. Cells were selected in the presence of Geneticin (Gibco; Cat# 11811-031), and lentiviruses carrying pLVX-Tight-Puro/STIL were used to infect the obtained Tet-On cells, which were further selected by puromycin (Gibco; Cat# A1113803).

### Generation of stable STIL-, FOXM1-, and HIF1α-knockdown cell lines

Stable knockdown cell lines were generated using lentiviruses carrying different shRNA against STIL, FOXM1, or HIF1α, which were infected into NCI-H1299, NCI-H2009, CL1-3 or CL1-5 cells. The cells were then maintained in media containing puromycin. All short hairpin RNA constructs were purchased from National RNAi Core Facility at Academia Sinica. The targeted sequences of various shRNAs used in this paper are listed in Additional file 1: Table S4.

### siRNA analysis

To knockdown the endogenous SAS6 expression, ON-TARGETplus siRNA SMARTpools (Dharmacon, GE Healthcare) was used. The sequences of siRNA and non-targeting siRNA control (si-Con) are listed in Additional file 1: Table S4. siRNA transfections were performed using Lipofectamine RNAiMAX (Invitrogen; Cat# 13778150) according to the manufacturer’s protocol.

### Cytoplasmic and nuclear fractionation

For the isolation of nuclear and cytoplasmic fractions, NE-PER Nuclear and Cytoplasmic Extraction Reagents (Thermo; Cat# 78833) were used according to the manufacturer’s protocol. Briefly, cells were washed with 1× ice-cold PBS buffer and lysed with the provided Cytoplasmic Extraction Reagent I and II. After centrifugation, the supernatant fraction (cytoplasmic extract) was collected. The insoluble pellet was resuspended in the provided Nuclear Extraction Reagent and incubated on ice. After centrifugation, the supernatant fraction was collected as the nuclear extract. The cytoplasmic and nuclear extracts were further analyzed by Western blot analysis.

### Cytoplasmic, membrane, nuclear soluble, chromatin-bound and cytoskeletal protein extracts

For the separation of cytoplasmic, membrane, nuclear soluble, chromatin-bound and cytoskeletal components, cells were lysed and fractionated using the Subcellular Protein Fractionation Kit (Thermo; Cat# 78840), according to the manufacturer’s protocol, which enables segregation and enrichment of proteins from five different cellular compartments. Briefly, the cultured cells were lysed by Cytoplasmic Extraction Buffer at 4 °C for 10 min. After centrifugation, the supernatant was collected as cytoplasmic extract, and the pellet was incubated with Membrane Extraction Buffer. After centrifugation, the supernatant was marked as membrane extract, and the pellet was further dissolved in Nuclear Extraction Buffer. After centrifugation, the supernatant was used as soluble nuclear extract, and the pellet continued to incubate with Nuclear Extraction Buffer containing CaCl_2_ and Micrococcal Nuclease. After centrifugation, the supernatant was collected as chromatin-bound nuclear extract. The recovered insoluble pellet was then extracted with the final reagent to isolate cytoskeletal proteins.

### Western blotting and immunoprecipitation (IP) analyses

To detect the protein expression, Western blotting analysis was performed. Briefly, the cells were lysed in RIPA buffer [43] with protease inhibitor cocktail (Roche; Cat# 1697498), and subjected to SDS-PAGE. After transfer, the membrane was probed with the indicated antibodies. To analyze the protein–protein interaction, an IP assay was conducted. After cell lysis, the protein lysates were incubated with the indicated antibodies at 4 °C for 1 h, and the samples were further reacted with protein A/G agarose beads overnight at 4 °C. The precipitated immune-complexes were washed and examined by Western blotting.

### Cell proliferation assay

The cell proliferation rate was determined using a Cell Counting Kit-8 (CCK-8) assay (Dojindo Molecular Technologies; Cat# CK04). Cells were seeded to a 96-well plate and incubated for the indicated times. After the CCK-8 reagent was added to cells, the cellular proliferation rate was measured by detection of the absorbance at 450 nm.

### Boyden chamber assay

In vitro cell migration and invasion assays were examined by Boyden chamber assay. For migration assay, cells suspended in the serum-free medium were seeded into the transwell (Falcon; Cat# 353504) and placed into the well containing medium supplemented with 10% FBS. After the incubation for 16 h, these migrated cells were fixed by methanol and stained by Giemsa solution (Sigma; Cat# GS500). For invasion study, the transwell was coated with matrigel prior to usage. The migrated or invaded cell numbers were counted by the ImageJ analysis software [88].

### Colony formation assay

For colony formation assay, the desired cells were seeded to a 6-well plate and incubated, with medium changes performed every 3 days. After incubation for 12 days, cells were stained by crystal violet. The numbers of colonies were counted using the ImageJ software.

### 3D migratory assay

To examine the 3D cell migration capability, cells were labeled with Cyto-IDTM Red (ENZO Life Sciences; Cat# ENZ-51037-K025) and seeded to 96-well culture plates that had been pre-coated with Matrigel (Corning; Cat# 356231). Cell motility was recorded every hour for 24 consecutive hours under a Leica DMI6000B fluorescence microscope (Leica Microsystems) and further analyzed with the MetaMorph 7.7.5 software (Molecular Devices).

### Immunocytochemistry (ICC) and immunofluorescent (IF) staining

The cells seeded on sterilized glass coverslips were fixed with 4% paraformaldehyde prior to treatment with 100% methanol. After incubation with blocking buffer (10% normal goat serum and 0.3% Triton X-100), the cells were treated with the indicated primary antibody overnight at 4 °C. The cells were washed with 1× PBST buffer, and further incubated with Alexa Fluor 568-conjugated (Invitrogen; Cat# A11031 (anti-mouse) or Cat# A11036 (anti-rabbit)) or Alexa Fluor 647-conjugated (Invitrogen; Cat# A32787) secondary antibodies and DAPI. Finally, the sections were washed extensively, and mounted. Images were obtained under confocal microscopy (Carl Zeiss; LSM 700 stage or LSM 780).

### qPCR

Total RNAs were extracted with the RNeasy Mini Kit (Qiagen; Cat# 74104) and converted to cDNAs using a SuperScript III first-strand synthesis system (Invitrogen; Cat# 18080-051). The gene expression was measured by qPCR analysis using the SYBR Green PCR Master Mix (Applied Biosystems; Cat# 4472908) on the 7500 Real-Time PCR system (Thermo Fisher Scientific). The *β-actin* mRNA was used as an internal control to normalize gene expression. The primers used in this assay are listed in Additional file 1: Table S4.

### Luciferase reporter assay

The promoter activities of the *SLUG*, *SOX2*, *NANOG*, *POU5F1,* and *STIL* gene promoters were measured using a Luciferase Assay System (Promega; Cat# E1500). Cells were co-transfected with the indicated promoter-luciferase construct and the indicated plasmids along with pEGFP-C1. After transfection, the cells were harvested and lysed in 1× Passive Lysis buffer. The luciferase activity was measured at 560 nm and the data were normalized by GFP intensity.

### Mouse line

Four-week-old male BALB/c NU mice (National Laboratory Animal Center, Taiwan) were used for the in vivo tumor formation and in vivo metastasis analyses. Mice used in our study were housed in SPF animal facility. All animal procedures were performed according to the guidelines and approved by the Institutional Animal Care and Use Committee of Academia Sinica.

### In vivo xenograft tumor formation assay

A total of 1 × 10^6^ cells were suspended in 1× PBS buffer and subcutaneously injected into the right flanks of BALB/c NU mice. The tumor size was measured every week, and the tumor volume was calculated according to the formula: volume = length × width^2^ × 0.52.

### In vivo metastasis assay

For the in vivo metastatic assay, STIL-knockdown CL1-5 cells were infected with lentivirus carrying luciferase and maintained in the presence of blasticidin (Invitrogen; Cat# 46-1120) to generate STIL-knockdown CL1-5 cells overexpressing luciferase. A total of 1 × 10^6^ cells were injected into BALB/c NU mice through the tail vein. After luciferin (PerkinElmer; Cat# 122799) was injected intraperitoneally, lung metastasis was monitored weekly with an in vivo imaging system (IVIS) (PerkinElmer; IVIS 200).

### Next-generation sequencing (NGS)

Total RNAs were extracted from CL1-0 and CL1-0 cells overexpressing GFP-STIL after 48 h Dox treatment using the RNeasy Mini Kit (Qiagen; Cat# 74104), and submitted to BioTools Company (Taiwan) for further NGS analysis. For the cDNA library preparations, the mRNAs were enriched using oligo dT beads, and then fragmented randomly in fragmentation buffer, followed by cDNA synthesis using random hexamers and reverse transcriptase. After first-strand synthesis, a custom second-strand synthesis buffer (Illumina) was added with dNTPs, RNase H and *Escherichia coli* polymerase I to generate the second strand by nick-translation. The cDNA library was available after a round of purification, terminal repair, A-tailing, ligation of sequencing adapters, size selection and PCR enrichment. Novaseq 6000 was used for the sequencing plate form, and read length was PE150. HTSeq v0.6.1 was conducted to count the reads numbers mapped to each gene. FPKM (Fragments Per Kilobase per Million) of each gene was calculated based on the length of the gene and reads count mapped to this gene. The data had been submitted to SRA database (BioProject ID: PRJNA799453). Finally, the hallmark gene sets of the gene set enrichment analysis (GSEA; https://www.gsea-msigdb.org/gsea/index.jsp) [89, 90] was used to identify the genes involved in the STIL-mediated potential pathways.

### ChIP-qPCR

To characterize the DNA–protein interaction, ChIP assay was performed using the MAGnify Chromatin Immunoprecipitation System (Invitrogen; Cat# 49-2024). Cells were fixed with 1% formaldehyde and their chromatins were sheared into the appropriate fragments by sonication. The chromatin fragments were incubated with the indicated antibodies including anti-STIL, anti-HIF1α, and anti-FOXM1 antibodiesy (Additional file 1: Table S3). After further washing, the cross-linked chromatin-protein complexes were treated by Reverse Crosslinking Buffer with proteinase K. The precipitated DNAs were isolated, purified, and subjected to qPCR analysis using the SYBR Green method (Applied Biosystems; Cat# 4472908) on the 7500 Real-Time PCR system (Thermo Fisher Scientific). The list of primer sequences is shown in Additional file 1: Table S4. Among them, primers that recognize *ARG2* (Arginase 2) promoter and *VEGF* (Vascular endothelial growth factor) promoter were served as the positive control for FOXM1 binding [91] and HIF1α binding [92], respectively. In contrast, primers for nearby 6.0 kb upstream region of the *SLUG* and *STIL* transcriptional start site were used for the unrelated control. An antibody against histone H3 trimethylated lysine 9 (H3-K9Me3) on the *SAT2* (Spermidine/Spermine N1-acetyltransferase family member 2) gene was used as the positive control for ChIP, and IgG was used as the negative control of antibody. The ChIP-qPCR signals were generated from three independent experiments followed by normalization to input signals as described [93, 94] and presented as the mean ± SD.

### Quantification and statistical analysis

Statistical analyses were performed using GraphPrism 6.01 and the data are presented as the mean ± standard derivation (SD) from at least three independent experiments. Statistical differences between two data sets were analyzed using the two-tailed paired or unpaired Student’s t test; *p < 0.05, **p < 0.01, and ***p < 0.001 were considered statistically significant. NS, not significant. To test the ability of a marker to distinguish cancers from non-malignant tissues, we performed Receptor Operating Characteristics (ROC) analysis and determined the area under the curve (AUC; values between 0.9–1 were considered excellent, between 0.8–0.9 were good, between 0.7–0.8 were fair, between 0.6–0.7 were poor, and between 0.5–0.6 represented failure). Kaplan–Meier survival curve analysis was used to examine the correlation between the expressing genes and prognosis, and the differences were estimated by the log-rank test. The hazard ratio was also assessed. Two-way ANOVA was used to analyze the differences of in vitro  cell proliferation rate, in vivo xenograft tumor formation, and in vivo metastasis. The Pearson correlation coefficient (R) was used to measure the correlation between two groups.

## Results

### STIL is up-regulated in patients with lung and various other types of cancers, and correlated with poor prognosis

**Fig. 1:**
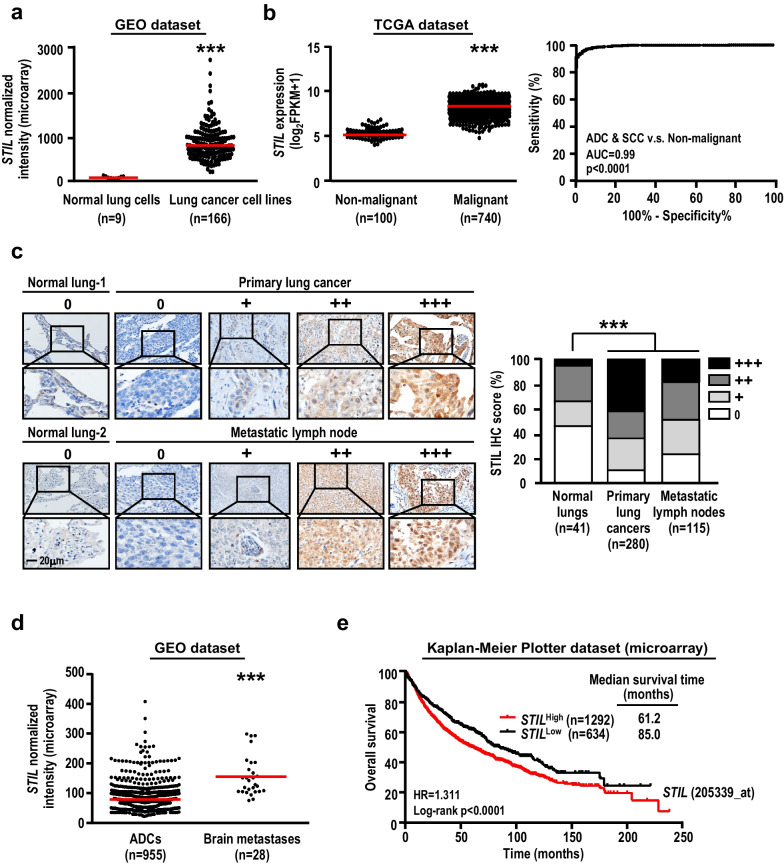
STIL is up-regulated in lung cancer patients and correlated with poor prognosis. **a** *STIL* expression determined by microarray analysis of 9 normal lung cell lines and 166 lung cancer cell lines derived from GEO. **b** *STIL* expression was determined by RNA sequencing of 100 solid tissue normal samples (referred to non-malignant lung tissues) and 740 ADCs and SCCs derived from TCGA, and the score for the AUC are shown (right panel). **c** STIL protein levels were analyzed by IHC analysis of 41 normal lung tissue samples, 280 primary lung cancers, and 115 paired metastatic lymph nodes (derived from the pool of 280 primary lung cancers). The normal lung tissues were used as non-malignant comparators for these lung cancers and metastatic lymph nodes. Representative IHC images of STIL staining are shown (left panel). Scale bar: 20 μm. IHC staining intensity was grouped as 0 (negative), + (weak), ++ (moderate), and +++ (strong), and the percentage distribution is depicted (right panel). **d** *STIL* expression was determined by microarray analysis of 955 lung ADCs and 28 brain metastases derived from lung ADCs. These data were derived from GEO. **e** Kaplan–Meier survival analysis of *STIL* gene expression was performed using microarray data of 1926 lung cancers derived from Kaplan–Meier Plotter. The median survival of the two groups is shown. Significance is determined by the log-rank test (p < 0.0001). The histologic subtypes of lung cancer in **b**–**e** are described in Additional file 1: Table S2. Data information: In **a**, **b** and **d**, the red lines indicate the median. In **a**–**d**, significance is determined by t-test (***p < 0.001)

Although centrosome amplification is frequently observed in human cancer [36], it is not yet known whether these centriolar/centrosomal proteins are involved in the pathogenic development of cancer. To begin addressing this, we first examined the T (tumor)/N (paired non-malignant) gene expression ratios for a number of centriolar/centrosomal genes in cDNA microarray data obtained from 163 lung cancer patients and their paired adjacent non-malignant lung tissues. The centriolar/centrosomal genes in Table 1 were selected on the basis of our and other groups’ studies [26–34, 42–49, 95, 96]. Most of the examined centriolar/centrosomal genes showed no difference or even a slight down-regulation between lung cancers and paired non-malignant tissues; however, the expression levels of *STIL*, *SASS6*, *SPICE1*, *PLK4*, *CEP295*, *POC5*, and *CEP152* displayed significantly high T/N ratios in lung cancer patients (Table 1). Among them, *STIL* showed the highest T/N ratio (3.5-fold increase), suggesting that it might play a unique and significant role in tumor development. A similar result was also obtained in lung cancer cell lines (Fig. 1a) and in RNA-seq data analysis of 100 non-malignant lung tissues and 740 lung cancer patients collected from the TCGA dataset (Fig. 1b, left panel). Importantly, *STIL* expression levels can be used to clearly distinguish lung cancers from non-malignant lung tissues, with an AUC value of 0.99 (Fig. 1b, right panel), suggesting that it could be useful for lung cancer detection.

**Table 1:** Centriolar/centrosomal genes expression (microarray)

Gene	Lung cancer patients			
	Tumor (n = 163)	Paired non-malignant (n = 163)	Ratio (T/N)	p-value
*STIL/MCPH7*	137.8	39.5	3.5	1.8E−29
*SASS6*	122.5	75.3	1.6	1.0E−19
*SPICE1*	184.1	116.7	1.5	1.1E−18
*PLK4*	57.8	37.7	1.5	2.3E−22
*CEP295/KIAA1731*	73.1	53.5	1.3	5.7E−08
*POC5*	212.4	164.4	1.2	3.6E−08
*CEP152/MCPH9*	27.4	24.1	1.1	8.1E−08
*CPAP/CENPJ/MCPH6*	111.7	104.6	1.0	4.3E−03
*CETN1*	27.3	26.6	1.0	8.8E−02
*RTTN*	141.7	139.4	1.0	1.9E−03
*CEP135/MCPH8*	81.7	81.6	1.0	5.6E−02
*CEP63*	128.9	144.0	0.9	1.3E−01
*POC1B*	376.0	425.2	0.9	3.8E−03
*CEP120*	401.9	507.8	0.8	3.1E−11

The microarray data of 163 paired lung cancer patients and their adjacent non-malignant lung tissues were derived from GEO

Ranking is based on the ratio between tumor (T) and paired non-malignant lung specimens (N)

Significance is determined by paired t-test

We performed further IHC analysis of normal lungs (n = 41), primary lung cancers (n = 280), and metastatic lymph nodes (n = 115) derived from the pool of 280 primary lung cancers. Compared with normal lungs, STIL protein intensity was significantly elevated in the primary lung cancers and metastatic lymph node groups (Fig. 1c). Since brain is one of the most common sites of distant metastasis of lung cancer, we also examined STIL expression in brain metastases derived from lung cancer. As shown in Fig. 1d, *STIL* mRNA level was significantly increased in brain metastases relative to primary lung cancers. More importantly, lung cancer patients with high levels of *STIL* usually displayed shorter overall survival than those with low levels of *STIL*, which were estimated from either microarray data (Fig. 1e) or RNA-seq data (Additional file 2: Fig. S1). Taken together, these data suggest that *STIL* high expression is correlated with poor prognosis.

Increased *STIL* mRNA levels were also observed in various other types of cancers, including breast, prostate, uterine, kidney, head and neck, liver, bladder, colon, and thyroid cancers (Additional file 1: Table S1a), where it was also associated with poor overall survival (Additional file 1: Table S1b). Furthermore, a high correlation was observed between the increased STIL expression level (*STIL*^high^) and shortened patient survival in lung ADCs and SCCs, indicating no bias toward a certain subtype of lung cancer (Additional file 1: Table S1b). Given that lung SCCs and lung ADCs were found to be the major cancer types with high-level *STIL* expression (Additional file 1: Table S1a), we focused our present efforts on lung cancer. Taken together, our data indicate that the mRNA and protein levels of STIL are up-regulated in lung and various other types of cancers, and that they are highly associated with poor prognosis.

### STIL depletion inhibits cancer cell proliferation, colony formation, and xenograft tumor formation

Given that STIL is up-regulated in lung cancer and associated with worse prognosis (Fig. 1), we first examined its oncogenic properties in two NSCLC cell lines, NCI-H1299 and NCI-H2009, which expressed high levels of *STIL* mRNA. Endogenous STIL was depleted in these two cell lines using lentivirus-based sh-RNAs (sh-STIL-1 and -2) and sh-Luc as a control (hereafter referred to as sh-Con) (Additional file 2: Fig. S2a, upper panel). Our results showed that STIL knockdown not only suppressed cell proliferation (Additional file 2: Fig. S2a, lower panel) and colony formation (Additional file 2: Fig. S2b), but also greatly inhibited the formation of xenograft tumors in nude mice (Additional file 2: Fig. S2c). Together, our results indicate that STIL depletion significantly blocks the oncogenic properties of lung cancer cells.

### STIL overexpression promotes cancer cell migration, invasion, and metastasis

**Fig. 2:**
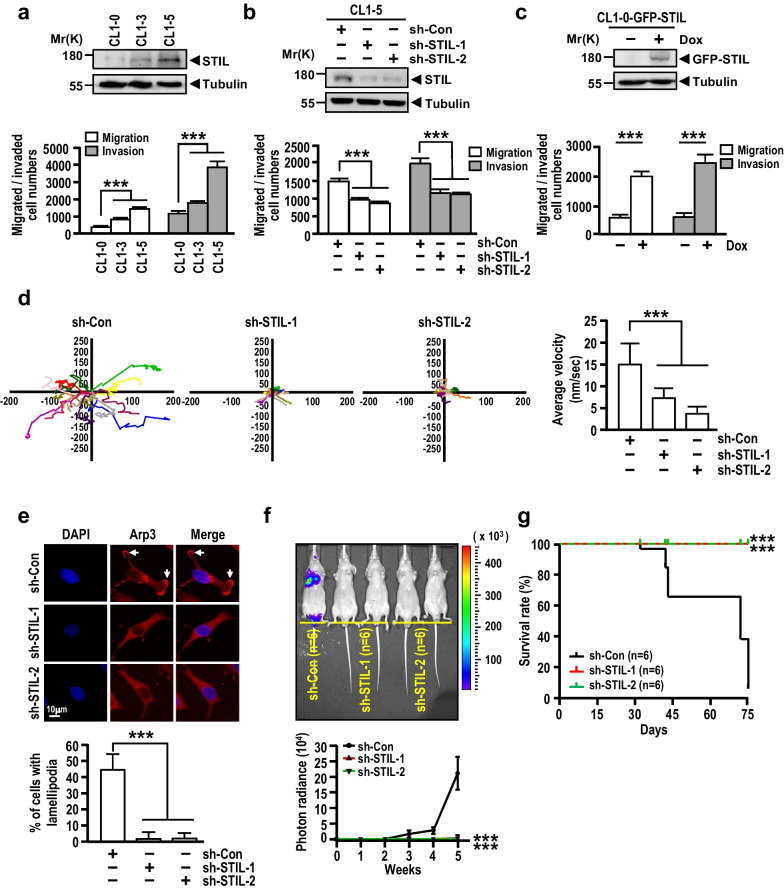
STIL overexpression promotes cancer cell migration and invasion in vitro and metastasis in vivo. **a**–**c** STIL protein levels (upper panel), cell migration, and invasion (lower panel) were analyzed by Western blotting and Boyden chamber assay in CL1-0, CL1-3, and CL1-5 cells (**a**), STIL-knockdown CL1-5 cells (**b**), or CL1-0 cells overexpressing GFP-STIL under Dox treatment for 48 h (**c**), respectively. **d** Cell migration ability was determined by a 3D migratory assay in STIL-knockdown CL1-5 cells, which were tracked by a time-lapse video microscopy system. Plotted tracks indicate the tracks of individual cells during a 24-h incubation period (n = 15); the velocity of the plotted tracks is shown (right panel). **e** The Arp-3 signal was determined by IF staining in STIL-knockdown CL1-5 cells (upper panel), and the cells with Arp-3 staining were quantified (lower panel). The white arrow indicates the Arp3 signal at the leading edge site. Scale bar: 10 μm. **f** In vivo metastasis was analyzed by tail-vein injection of STIL-knockdown CL1-5 cells overexpressing luciferase into c-nude mice. Bioluminescence images were obtained by an IVIS system at day 14 (upper panel) and the lung bioluminescence was quantified (lower panel) (n = 6 per group). Significance is determined by two-way ANOVA (***p < 0.001). **g** Survival time of c-nude mice given tail-vein injection of STIL-knockdown CL1-5 cells (n = 6 per group). Significance is determined by the log-rank test (***p < 0.001). Data information: Statistical data in **a**–**e** represent the mean ± SD (n = 3 independent experiments). Significance is determined by t-test (***p < 0.001)

Because STIL is also elevated in metastatic cancers (Fig. 1c, d), we next examined whether excess STIL could affect lung cancer cells migration and invasion. The human lung adenocarcinoma cell lines, CL1-3 and CL1-5, which are derived from the parental CL1-0 and possess progressive migration and invasiveness capabilities [84], were used to examine the oncogenic role of STIL. Intriguingly, an increase STIL level was found to be correlated with the progressive migration and invasiveness capabilities of these cells (Fig. 2a). To investigate whether the increased expression of STIL is indeed associated with enhanced cell migration and invasion activities, we knocked down STIL expression in CL1-5 cells, and examined the effects of this change. As shown in Fig. 2b, STIL depletion led to a marked decrease in the migration and invasion abilities of CL1-5 cells. Similar results were also observed in STIL-knockdown NCI-H1299 (Additional file 2: Fig. S3a, left panel) and CL1-3 cells (Additional file 2: Fig. S3a, right panel), suggesting that the effects of STIL knockdown on migration and invasion are independent of the lung cancer cell line used. Conversely, overexpression of GFP-STIL (triggered by Dox treatment) enhanced the migratory and invasive capabilities of CL1-0 cells (Fig. 2c). Furthermore, we observed no significant differences of cell growth between sh-Con and sh-STIL-treated CL1-5 cells (Additional file 2: Fig. S3b, left panel) or between Dox treated and untreated CL1-0-GFP-STIL cells (Additional file 2: Fig. S3b, right panel) after a 16-h incubation time (a time period used for monitoring cell migration and invasion), thus excluding the possibility that the effects of STIL on migration and invasion are resulted from the perturbation of cell growth. A further 3D assay of cancer cell migration confirmed that STIL knockdown in CL1-5 cells significantly inhibited the cell migration velocity (Fig. 2d). Consistent with these findings, we found that Arp3 (a lamellipodial marker) was significantly reduced at the leading edge sites of lamellipodia in sh-STIL-treated CL1-5 cells (Fig. 2e). Together, our findings indicate that STIL promotes cancer cell migration and invasion in in vitro cultured cells.

We next examined the role of STIL in metastasis using bioluminescent in vivo imaging analysis. As shown in Fig. 2f, sh-Con-treated CL1-5 cells developed lung metastases as early as 2 to 3 weeks after injection, while no or very low lung metastasis was detected in sh-STIL-treated CL1-5 cells even at 5 weeks post-injection. Mice bearing sh-Con cells started to die at day 32, and the survival rate decreased to < 15% at day 75 (Fig. 2g). In contrast, mice injected with sh-STIL-treated CL1-5 cells showed 100% survival at day 75. Consistent with this finding, lung cancer patients with high levels of STIL show a poor survival rate (Fig. 1e, Additional file 2: Fig. S1). Taken together, our data strongly support the idea that STIL modulates lung cancer metastasis in vivo and its high-level expression is associated with poor survival of lung cancer patients.

Centrosome amplification has been shown to promote the invasive phenotype in a three-dimensional culture system [97], and overexpression of STIL leads to centriole amplification [42–44]. We next investigated whether STIL-induced centriole amplification is associated with their migration and invasion abilities. Toward this end, overexpression of STIL after Dox induction in NCI-H1299-based GFP-STIL-inducible cells were pretreated with si-SASS6 RNA to block the centriole amplification, and their migratory and invasive abilities were examined (Additional file 2: Fig. S4a). SAS6 was previously reported to play an essential role in centriole duplication, whose overexpression induces centriole amplification [98]. We therefore selected the si-SASS6 to block centriole amplification. As expected, overexpression of STIL induced centriole amplification (> 4 centrioles), while co-treated the cells with si-SASS6 markedly decreased centriole numbers (Additional file 2: Fig. S4b). Furthermore, we found that the migrated/invaded cell numbers were significantly reduced in the Dox- and si-SASS6-treated cells harboring excess STIL with perturbing centriole amplification by si-SASS6 (Additional file 2: Fig. S4a). Interestingly, the remaining migrated/invaded cell numbers in the Dox- and si-SASS6-treated cells were still higher than those in non-Dox- and si-SASS6-treated cells (Additional file 2: Fig. S4a). Since our experiments did not knockout the *SASS6* gene to completely block excess STIL-mediated centriole amplification due to acentriolar cells are non-viable in the presence of p53, we thus can’t exclude the possibility of centrosome amplification in tumorigenesis. Nevertheless, these results imply that in addition to centriole amplification, overexpression of STIL could induce additional mechanism(s) to promote migration and invasion.

### STIL enters into the nucleus and activates the EMT pathway to promote cancer cell migration and invasion

**Fig. 3:**
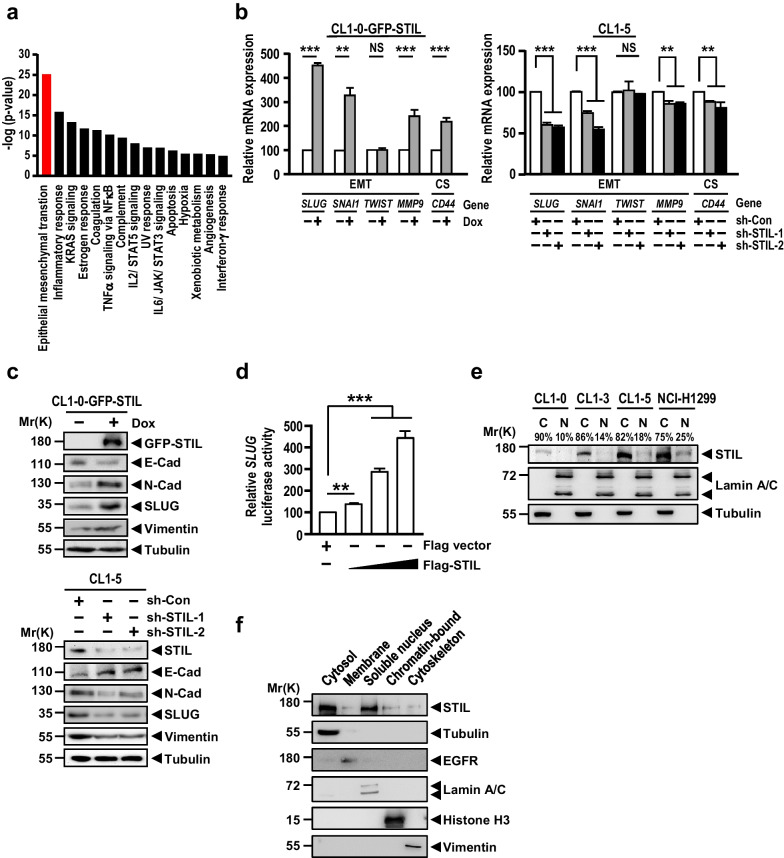
STIL activates the EMT signaling pathway to promote cancer cell migration and invasion. **a** The major biological functions analyzed by GSEA analysis of the transcriptome differences between CL1-0 cells and CL1-0 cells overexpressing GFP-STIL under Dox treatment for 48 h. The red bar indicates the most significant biological function. **b** The relative mRNA levels of EMT-TFs (*SLUG*, *SNAI1*, and *TWIST*), the EMT-regulator *MMP9*, and the CS marker *CD44*, as measured by qPCR method in Dox inducible CL1-0 cells overexpressing GFP-STIL (left panel) and in STIL-knockdown CL1-5 cells (right panel). **c** The protein levels of EMT regulators were analyzed by Western blotting in Dox inducible CL1-0 cells overexpressing GFP-STIL (upper panel) and STIL-knockdown CL1-5 cells (lower panel). Tubulin served as loading control. **d** *SLUG* promoter activity was measured by reporter assay in HEK293T cells transiently transfected with a pGL3/*SLUG* promoter-luciferase plasmid and the indicated constructs. **e** STIL protein levels in cytoplasmic (C) and nuclear (N) fractions analyzed by Western blotting in the indicated cells. Lamin A/C and tubulin were used as nuclear and cytoplasmic markers, respectively. The percentage of subcellular distribution is also shown. **f** STIL protein levels derived from the subcellular fractions of cytosol, membrane, nuclear-soluble, chromatin-bound, and cytoskeleton in NCI-H1299 cells were analyzed by Western blotting. Tubulin, EGFR, lamin A/C, histone H3 and vimentin were used as cytoplasmic, membrane, nuclear-soluble, chromatin-bound, and cytoskeletal markers, respectively. Data information: Statistical data in **b** and **d** represent the mean ± SD (n = 3 independent experiments). Significance is determined by t-test (NS, not significant; **p < 0.01; ***p < 0.001)

To investigate the possible mechanism(s) underlying STIL-mediated metastasis, we used next-generation sequencing to examine the transcriptome differences in CL1-0 and CL1-0-based GFP-STIL-inducible cells. The result of GSEA analysis showed that STIL-modulated genes were involved in multiple cancer pathways, including the EMT process, hypoxia, and many other signaling pathways (Fig. 3a). Accordingly, we used qPCR analysis to examine the effects of STIL on EMT and CS. As shown in Fig. 3b (left panel), excess STIL induced upregulation of the mRNAs encoding a core set of EMT-transcription factors (*SLUG* and *SNAI1*, but not *TWIST*), the EMT-mediator *MMP9*, and the CS marker *CD44* in CL1-0 cells. Conversely, these genes (with the continued exception of *TWIST*) were downregulated in sh-STIL-treated CL1-5 cells (Fig. 3b, right panel).

Considering that EMT-related genes displayed the most significant changes after STIL induction (Fig. 3a), we examined the expression level of target proteins that are involved in the EMT signaling pathway. CL1-0 cells overexpressing GFP-STIL exhibited upregulation of mesenchymal marker proteins (N-Cadherin, SLUG, and Vimentin) and downregulation of an epithelial marker protein (E-Cadherin) (Fig. 3c, upper panel), whereas the opposite effects were observed in sh-STIL-treated CL1-5 cells (Fig. 3c, lower panel). Since SLUG was reported to be a key transcription factor for EMT [99, 100], we hypothesized that STIL, in addition to its known centriolar role, could serve as an activator to promote *SLUG* upregulation. To examine this possibility, we tested whether STIL could activate *SLUG* promoter-driven luciferase activity. As shown in Fig. 3d, STIL significantly activated *SLUG* promoter-driven luciferase activity in a dose-dependent manner, suggesting that STIL regulates *SLUG* expression at the transcriptional level. Accordingly, we proposed that a subset of STIL might translocate into the nucleus to activate *SLUG* expression. The nuclear (N) and cytosolic (C) fractions of indicated cells were isolated and analyzed by Western blotting. Our results showed that while STIL is mainly present in the cytoplasmic fraction (75–90%), a proportion of STIL protein (10–25%) is clearly detectable in the nuclear fraction (Fig. 3e). In addition, the protein levels of nuclear STIL (Fig. 3e) are correlated with the total STIL protein levels of their corresponding cells (Additional file 2: Fig. S5a). Consistently, the nuclear fraction of STIL was increased in CL1-0 overexpressing GFP-STIL under Dox treatment (Additional file 2: Fig. S5b, right panel) compared with the parental CL1-0 cells (24% versus 11%) (Additional file 2: Fig. S5b, left panel).

Given that a small amount of STIL can be found in the nuclear fraction, we further examined whether STIL is chromatin-bound. The cell extracts from NCI-H1299 cells were fractionated into cytoplasmic, membrane, nuclear-soluble, chromatin-bound, and cytoskeletal components. As shown in Fig. 3f, STIL was mainly detected in cytosol, soluble nuclear extract, and chromatin-bound fraction, but less in membrane and cytoskeletal fractions. We further examined the nuclear and cytoplasmic distribution of STIL in the same clinical specimens used in Fig. 1c. As shown in Additional file 2: Fig. S5c, STIL protein levels in both cytoplasm and nucleus were significantly up-regulated in primary lung cancers compared with the normal lung tissues. Collectively, our data thus suggest that a subset of STIL could enter the nucleus, bind to the chromatin, and upregulate factors involved in EMT pathway.

### STIL associates with FOXM1 to enhance its transcriptional activity and consequently regulates tumor metastasis and stemness

We next asked: How does STIL upregulate EMT-transcription factors (EMT-TFs)? Because no known DNA-binding domain has been found in the STIL protein, we hypothesized that STIL may serve as a coactivator that associates with a transcriptional factor to regulate *SLUG* gene expression. To screen for potential STIL-associated transcription factors that may be involved in the EMT pathway, we performed a bioinformatic search of microarray data from lung cancer cell lines (E-MTAB-37 derived from EBI). We first identified the STIL-correlating genes (381 genes) that showed a Pearson’s correlation coefficient (R) > 0.55, and the Human Transcription Factors database [101] was applied to narrow down the genes that belong to the transcriptional factors and also possess the ability to directly drive EMT (Additional file 2: Fig. S6a, b). Our analysis identified the *FOXM1* gene (Additional file 2: Fig. S6a). The correlation of *STIL* and *FOXM1* expression in clinical specimens was further analyzed by the Pearson’s correlation coefficient and showed a strong correlation (R = 0.87) (Additional file 2: Fig. S6c, upper panel). FOXM1 was reported as an important cell cycle transcription factor involved in tumor progression [102]. To investigate whether a similar correlation exists between STIL and other cell cycle regulators, we used the same cohorts and examined the correlation between *STIL* and *CCND1* (Cyclin D1), a G1 phase regulator of the cell cycle. The result showed that the correlation coefficient between *STIL* and *CCND1* was − 0.217 (Additional file 2: Fig. S6c, lower panel), suggesting the strong correlation of *STIL* and *FOXM1* is unique in lung cancer.

**Fig. 4:**
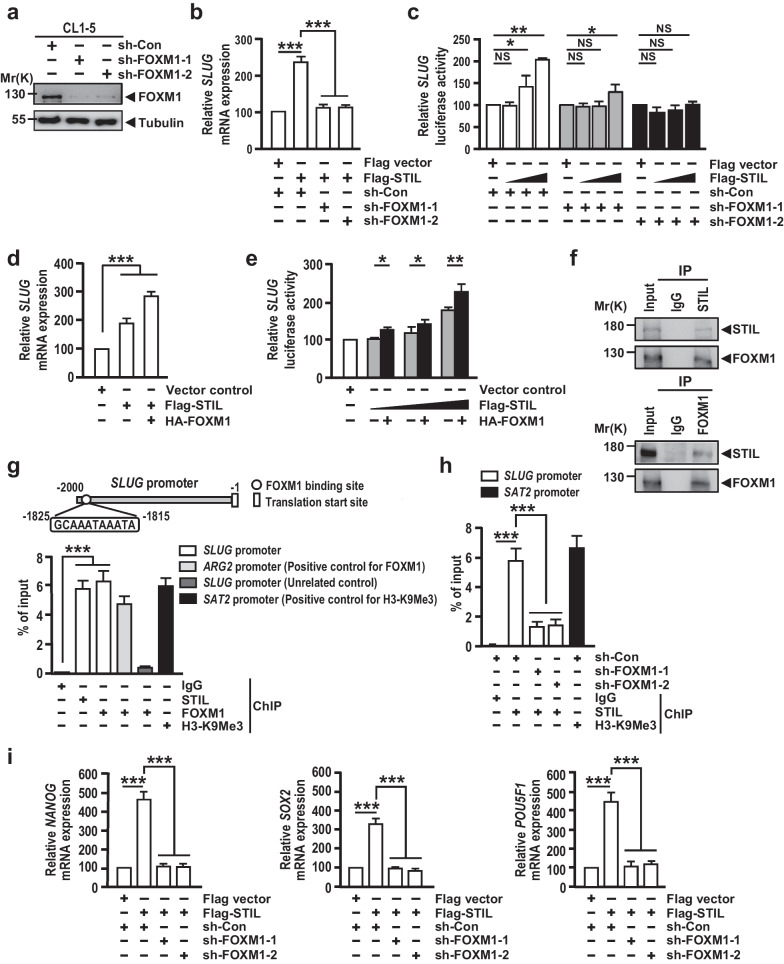
STIL associates with FOXM1 to enhance FOXM1-mediated transcriptional activity. **a** FOXM1 protein levels were analyzed by Western blotting in FOXM1-knockdown CL1-5 cells. Tubulin served as loading control. **b**–**e** *SLUG* mRNA levels were measured by qPCR method (**b** and **d**) and the *SLUG* promoter-driven luciferase activity was determined by reporter assay (**c** and **e**) in FOXM1-knockdown CL1-5 cells transiently transfected with indicated constructs (**b** and **c**) and CL1-0 cells transiently transfected with the indicated constructs (**d** and **e**). **f** The endogenous association between STIL and FOXM1 in CL1-5 cells was analyzed by co-IP analysis and Western blotting using the indicated antibodies. **g** The potential FOXM1 DNA-binding sites within the *SLUG* promoter (upper panel), and the binding of STIL and FOXM1 to the *SLUG* promoter were analyzed by ChIP-qPCR assay (lower panel) in CL1-5 cells. *ARG2* promoter was served as the positive control for FOXM1-binding, and the region of 6.0 kb upstream of *SLUG* transcriptional start site within *SLUG* promoter was used as the unrelated control. **h** The binding of STIL to the *SLUG* promoter was examined by ChIP-qPCR assay in FOXM1-knockdown CL1-5 cells. **i** The mRNA levels of *NANOG*, *SOX2*, and *POU5F1* were measured by qPCR method in FOXM1-knockdown CL1-5 cells transiently transfected with the indicated constructs. Data information: In **g** and **h**, Methylation of histone H3 (H3-K9Me3) on *SAT2* gene was used as a positive control and IgG as a negative control for the ChIP-qPCR assay. Statistical data in **b**–**e** and **g**–**i** represent the mean ± SD (n = 3 independent experiments). Significance is determined by t-test (NS, not significant; *p < 0.05; **p < 0.01; ***p < 0.001)

Interestingly, FOXM1 was reported to promote EMT by directly binding to the *SLUG* promoter [103]. We thus hypothesized that STIL may serve as a FOXM1-associated protein to upregulate *SLUG* expression. Accordingly, we tested the effect of FOXM1 on STIL-induced *SLUG* expression. Two CL1-5-based FOXM1 knockdown clones (sh-FOXM1-1 and -2) and their corresponding control (sh-Con) were generated using a lentivirus-based shRNA approach (Fig. 4a). Our results showed that the *SLUG* mRNA was increased in cells overexpressing Flag-STIL, but this effect was impaired in sh-FOXM1-treated cells (Fig. 4b). To further validate this result, we tested the effect of STIL on *SLUG* promoter activity. Depletion of FOXM1 led to the decreased *SLUG* promoter activity (Additional file 2: Fig. S6d), consistent with previous report [103]. Furthermore, overexpression of STIL increased the *SLUG* promoter-driven luciferase activity in a dose-dependent manner (Fig. 4c), while this enhancement effect could be blocked by FOXM1 depletion (Fig. 4c). Interestingly, FOXM1 overexpression could synergistically enhance not only STIL-induced *SLUG* mRNA expression (Fig. 4d) but also *SLUG* promoter-driven luciferase activity (Fig. 4e). Together, our results suggest that FOXM1 is required for STIL-induced *SLUG* upregulation.

We next examined whether STIL associates with FOXM1 in vivo. Our co-immunoprecipitation (co-IP) experiments showed that endogenous FOXM1 could form a complex with STIL, as visualized using either anti-STIL or anti-FOXM1 antibodies (Fig. 4f). The STIL-FOXM1 complex was found not only in the cytoplasm, but also in the nucleus (Additional file 2: Fig. S6e). The association of FOXM1 and STIL was further validated by exogenous expression of GFP-STIL and HA-FOXM1 in HEK293T cells (Additional file 2: Fig. S6f). We screened 2500 bp region of *SLUG* promoter with the canonical RYAAAYA Forkhead binding motifs (FKH motif; where R is a purine and Y is a pyrimidine) [104, 105], and identify a region (− 1825 to − 1815 bp) carrying two overlapping putative FOXM1 binding motifs (GCAAATAAATA; Fig. 4g) in the *SLUG* promoter. We then conducted the quantitative ChIP-qPCR assay to test whether the FOXM1-STIL complex could bind to the *SLUG* promoter. Our result showed that both of STIL and FOXM1 bound to the *SLUG* promoter (Fig. 4g). Notably, the association of both STIL (Fig. 4h) and FOXM1 (Additional file 2: Fig. S6g) with the *SLUG* promoter was reduced in sh-FOXM1-treated cells. Reciprocally, the reduced *SLUG* promoter-binding activity of FOXM1 was observed in STIL-depleted CL1-5 cells, suggesting that STIL enhances the transcriptional activity of FOXM1 by increasing its promoter binding affinity (Additional file 2: Fig. S6h). Since FOXM1 directly interacts with the *SLUG* promoter [103] and STIL does not harbor any known DNA-binding domains, we hypothesize that STIL acts as a transcriptional coactivator of FOXM1 to promote *SLUG* expression.

Given that the CS core transcription factors (e.g., SOX2, OCT4/*POU5F1*, and NANOG) were reported to be directly regulated by FOXM1 [106], we tested whether STIL regulates the expression of these CS core genes in a FOXM1-dependent manner. As expected, overexpression of STIL significantly increased the mRNA expression levels of *NANOG*, *SOX2*, and *POU5F1*; however, these STIL-induced upregulations were suppressed upon FOXM1 depletion (Fig. 4i). Further studies demonstrated that STIL depletion affects the expression of these FOXM1-driven genes: overexpression of FOXM1 significantly increased these CS gene promoter-driven luciferase activities (e.g. *SLUG, NANOG*, *SOX2* and *POU5F1*) in a dose-dependent manner, while knockdown of endogenous STIL reduced FOXM1-induced gene activation (Additional file 2: Fig. S6i). Together, our findings suggest that STIL associates with FOXM1 to enhance the FOXM1-modulated CS.

FOXM1 has also been reported to play an important role in cell cycle regulation that controls the expression of many genes required for G1/S and G2/M transition [107, 108]. To investigate whether STIL might modulate FOXM1-driven cell cycle related genes [109], the gene expression levels of the regulators for G1/S transition, such as *CCND1*, *SKP2* (S-Phase Kinase Associated Protein 2) and *CDC25A* (Cell Division Cycle 25A), the components of G2/M phase progression including *CCNB1* (Cyclin B1), *CCNB2* (Cyclin B2), *CDK1* (Cyclin Dependent Kinase 1) and *PLK1* (Polo Like Kinase 1), and the activators of mitotic entry, such as *AURKA* (Aurora kinase A), *AURKB* (Aurora kinase B), *CEBPB* (CCAAT/enhancer-binding protein beta) and *BUBR1/BUB1B* (Budding uninhibited by benzimidazoles 1 homolog beta), were examined. Two CL1-5-based FOXM1 knockdown clones and their corresponding control cells overexpressing Flag vector or Flag-STIL were generated (Additional file 2: Fig. S7a). As expected, FOXM1 deletion resulted in the decreased mRNA expression including *SKP2*, *CDC25A*, *CCNB1*, *CCNB2*, *CDK1*, *PLK1*, *AURKA*, *AURKB* and *BUBR1* (Additional file 2: Fig. S7b). Overexpression of STIL significantly increased the expression of the above genes; however, these STIL-induced upregulations were inhibited upon FOXM1 depletion. In contrast, FOXM1 knockdown or overexpression of STIL did not affect *CCND1* and *CEBPB* mRNA (Additional file 2: Fig. S7c). The reason is not clear. Collectively, our findings suggest that the association of STIL with FOXM1 could upregulate some FOXM1-modulated genes involved in cell cycle regulation, such as *SKP2*, *CDC25A*, *CCNB1*, *CCNB2*, *CDK1*, *PLK1*, *AURKA*, *AURKB,* and *BUBR1*, but not *CCND1* and *CEBPB*.

### The interaction between FOXM1 and STIL is required for the STIL-FOXM1 axis-mediated tumorigenic abilities

**Fig. 5:**
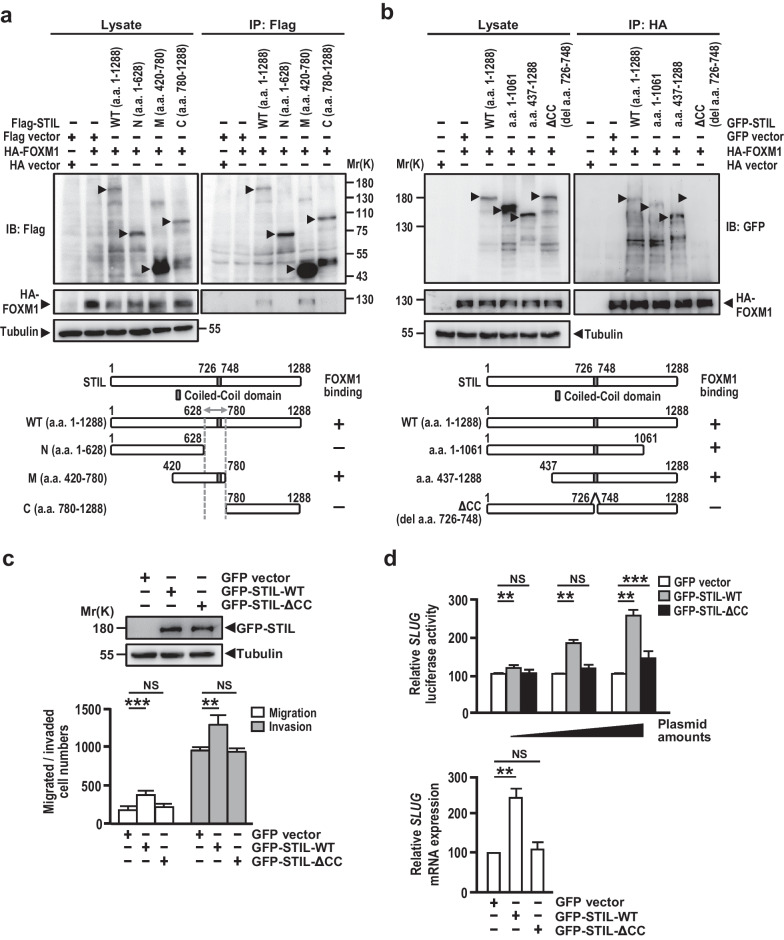
The interaction of FOXM1 with STIL is required for STIL-induced tumorigenic abilities. **a** HEK293T cells were transiently co-transfected HA-FOXM1 with full-length or various truncated Flag-STIL mutants including STIL-N (a.a. 1–628), STIL-M (a.a. 420–780) and STIL-C (a.a. 780–1288) as indicated. Protein complexes were immunoprecipitated using anti-Flag antibody and analyzed by Western blotting using indicated antibodies. **b** HEK293T cells were transiently co-transfected HA-FOXM1 with the full length or various truncated GFP-STIL mutants (a.a. 1–1061, a.a. 437–1288, and the deleted coiled-coil domain (ΔCC)). Protein complexes were immunoprecipitated using anti-HA antibody and analyzed by Western blotting. **c**, **d** NCI-H1299 cells were transiently transfected with GFP-STIL-WT or GFP-STIL mutant (ΔCC), and the expressed proteins were analyzed by Western blotting (**c**, upper panel). Cell migration and invasion abilities were analyzed by Boyden chamber assay (**c**, lower panel), while the *SLUG* promoter activity was measured by reporter assay (**d**, upper panel) and the *SLUG* mRNA level (**d**, lower panel) was measured by qPCR method. Data information: Statistical data in **c**, **d** represent the mean ± SD (n = 3 independent experiments). Significance is determined by t-test (NS, not significant; **p < 0.01; ***p < 0.001)

To validate the importance of the association of STIL with FOXM1 in STIL-mediated tumorigenic functions, we have mapped the FOXM1-interacting region of STIL. Our co-IP results showed that HA-FOXM1 forms complexes with full-length wild type STIL (a.a. 1–1288) and STIL-M (a.a. 420–780), but not STIL-N (a.a. 1–628) or STIL-C (a.a. 780–1288), implying that the region of STIL between a.a. 628 to 780 is responsible for FOXM1-binding (Fig. 5a). Because the coiled-coil domain (a.a 726–748) of STIL [110] was reported to be located within this region, we examined whether the deleted coiled-coil domain mutant of STIL (ΔCC) impairs its FOXM1-binding ability. As shown in Fig. 5b, the full-length STIL and two STIL truncated mutants (a.a. 1–1061 and a.a. 437–1288) containing the coiled-coil domain are coimmunoprecipitated with FOXM1, while the STIL mutant missing the coiled-coli domain (ΔCC) does not. We next examined the tumorigenic effects of the STIL mutant (ΔCC) on migration/invasion and *SLUG* gene expression. Our results showed that the STIL mutant (ΔCC) impaired its abilities to enhance cellular migration/invasion (Fig. 5c) and *SLUG* gene activation (Fig. 5d), suggesting that the interaction of FOXM1 with STIL is important for STIL-mediated tumorigenic abilities. Together, our data support a model wherein STIL acts as a coactivator that complexes with FOXM1 to upregulate the FOXM1-mediated downstream genes involved in metastasis, CS, and cell cycle.

### STIL expression is induced by HIF1α under hypoxia

We next explored why STIL is up-regulated in lung cancer. We first examined the *STIL* DNA copy number using single-nucleotide polymorphism (SNP) array data (Additional file 2: Fig. S8a) and the DNA methylation status of *STIL* using 450K methylation array data (Additional file 2: Fig. S8b). No significant difference was observed among normal lungs, lung cancer cell lines, and lung cancer specimens in either datasets.

**Fig. 6:**
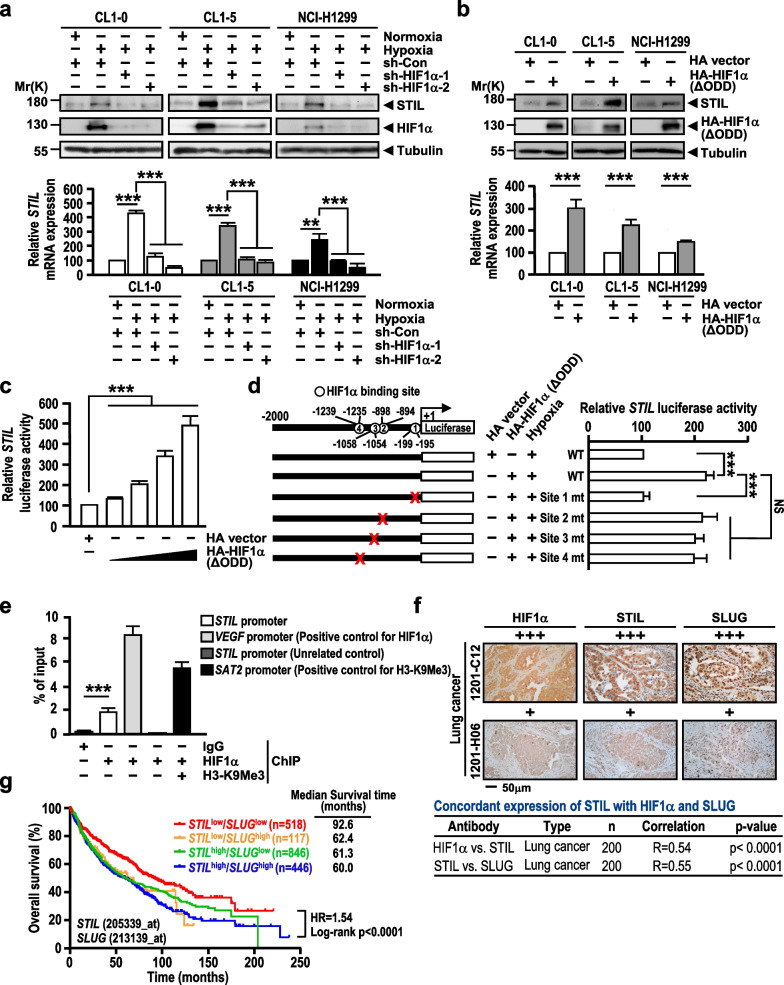
STIL expression is induced by HIF1α under hypoxia. **a** STIL protein (upper panel) and mRNA levels (lower panel) were analyzed by Western blotting and qPCR method, respectively, in the indicated cells under normoxic (20% O_2_) or hypoxic (1% O_2_) conditions, or in HIF1α-knockdown cells under hypoxia. **b** STIL protein (upper panel) and mRNA levels (lower panel) were analyzed in cells transiently transfected with the indicated constructs under normoxia. **c** *STIL* promoter activity was measured by reporter assay in HEK293T cells transiently transfected with the pGL3-*STIL* promoter-luciferase plasmid and the indicated constructs under the normoxic condition. **d** *STIL* promoter activity was measured by reporter assay in CL1-0 cells transiently transfected with pGL3-*STIL* promoter-driven luciferase constructs encoding wild-type HIF1α, HIF1α DNA-binding site mutants, and/or HA-HIF1α (ΔODD) under hypoxia. **e** The binding of HIF1α to the *STIL* promoter was analyzed by the ChIP-qPCR assay in CL1-5 cells under hypoxia. *VEGF* promoter was served as the positive control for HIF1α binding, and the region of 6.0 kb upstream of *STIL* transcriptional start site within *STIL* promoter was used as the unrelated control. Methylation of histone H3 (H3-K9Me3) on *SAT2* gene was used as a positive control and IgG as a negative control for the ChIP-qPCR assay. **f** Clinical correlations between STIL, HIF1α, and SLUG in 200 lung cancer specimens were analyzed by IHC and the data were assessed using Pearson’s correlation. Scale bar: 50 μm. **g** Overall survival of 1927 lung cancer patients stratified by *STIL* and *SLUG* expression was determined by Kaplan–Meier analysis. The median survival of the four groups is shown. Significance is determined by the log-rank test (p < 0.001). Data information: Statistical data in **a**–**e** represent the mean ± SD (n = 3 independent experiments). Significance is determined by t-test (NS, not significant; **p < 0.01; ***p < 0.001)

Hypoxia is an important micro-environmental characteristic that activates EMT-TFs and the HIF-mediated pathway during tumor metastasis [111]. The hypoxia pathway was also noted in our GSEA analysis of the STIL-regulated transcriptome (Fig. 3a), and four HIF1α DNA-binding sites ([A/G]CGTG) were identified in the *STIL* promoter (Fig. 6d). Interestingly, we found that an increase HIF1α level was accompanied by the elevated STIL expression (Fig. 2a) in CL1-0, CL1-3 and CL1-5 cells under the normoxic condition (Additional file 2: Fig. S8c). We thus investigated whether STIL is induced under the hypoxic condition. Figure 6a shows that the protein and mRNA levels of STIL were up-regulated in CL1-0, CL1-5, and NCI-H1229 cells under hypoxia, but that HIF1α depletion dramatically diminished the ability of hypoxia to induce STIL at the protein (Fig. 6a, upper panel) and mRNA (Fig. 6a, lower panel) levels in all three lung cancer lines. HIF1α, which is the major transcription factor in the cellular response to low oxygen, is easily degraded in normoxia. We thus examined whether overexpression of HIF1α (ΔODD), an HIF1α mutant that lacks the oxygen-degradation domain could induce STIL expression under normoxia. As shown in Fig. 6b, we observed increases of STIL at both the protein and mRNA levels in cells overexpressing HIF1α (ΔODD) under the normoxic condition. Consistent with this finding, HIF1α (ΔODD) overexpression could activate *STIL* promoter-driven luciferase activity in a dose-dependent manner under normoxia (Fig. 6c). Given that a small portion of STIL can be detected in nucleus (Fig. 3e) and STIL is upregulated by HIF1α (Fig. 6a), we further examined the effect of hypoxia on STIL nuclear localization. As shown in Additional file 2: Fig. S8d, the endogenous nuclear STIL was slightly increased under the hypoxic condition compared with that of normoxia (20% versus 11%). The ICC result further showed that HIF1α became stable and accumulated in the nucleus under hypoxia, and led to the increased FOXM1 in the nucleus (Additional file 2: Fig. S8e). Furthermore, hypoxia seems to partially promote the nuclear localization of GFP-STIL (Additional file 2: Fig. S8e), which may reflect with an increase of nuclear GFP-STIL protein detected by Western blotting under hypoxia (36% versus 29%) (Additional file 2: Fig. S8f). Together, our results indicate that HIF1α is an upstream factor that regulates STIL expression.

Since we identified four HIF1α consensus DNA-binding sites (Fig. 6d) in the *STIL* promoter (site1: nts − 195 to − 199; site2: − 894 to − 898; site3: − 1054 to − 1058; and site4: − 1235 to − 1239), we then generated mutations in these four sites ([A/G]CGTG mutated to [A/G]CTGT) and examined which site is responsible for HIF1α binding. Our results showed that the mutation within nts − 195 to − 199 (site1) dramatically inhibited HIF1α-induced luciferase activation in cells overexpressing HIF1α (ΔODD) under hypoxia (Fig. 6d). A similar effect was also observed in HIF1α (ΔODD)-overexpressing cells under normoxia (Additional file 2: Fig. S8g). To examine whether HIF1α directly binds to the *STIL* promoter, we performed ChIP-qPCR assay in CL1-5 cells that were pre-treated with hypoxia (to stabilize the HIF1α protein level). Our result showed that HIF1α binds to the *STIL* promoter under the hypoxic condition (Fig. 6e). Collectively, our findings suggest that HIF1α directly binds to the *STIL* promoter at the site1 region (nts − 195 to − 199) to drive STIL expression under hypoxia.

We next performed IHC analysis to assess the clinical relevance of STIL in relation to HIF1α and SLUG. Our results showed that the HIF1α intensity was positively correlated with the expression of STIL (Pearson’s co-efficient, R = 0.54) in lung cancer specimens (Fig. 6f). STIL expression was similarly associated with SLUG in the same patient cohort (R = 0.55, Fig. 6f). Intriguingly, the concordant expression of STIL with SLUG displayed an especially high correlation in patients with metastatic lymph nodes (R = 0.76, Additional file 2: Fig. S8h), suggesting that there is a strong association of the HIF1α-STIL-SLUG axis in lung cancer specimens. We thus evaluated the clinical application of the *STIL*-*SLUG* axis to predict survival among lung cancer patients. We found that patients with *STIL*^high^ and *SLUG*^high^ were associated with poor patient survival (60.0 months) (Fig. 6g), whereas *STIL*^low^ and *SLUG*^low^ patients exhibited prolonged survival (92.6 months). This suggests that the *STIL*-*SLUG* axis could be a useful prognostic marker for the survival rate of lung cancer patients.

## Discussion

**Fig. 7:**
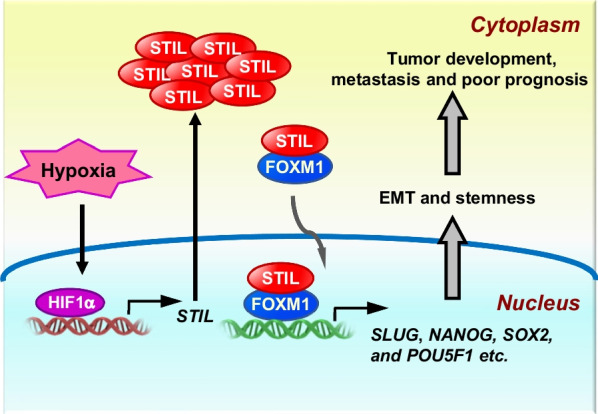
Schematic diagram illustrates the molecular mechanism how STIL contributes to tumor development via the FOXM1-mediated transcriptional activation of target genes under hypoxia. HIF1α becomes stable under hypoxia in cancer, and translocates to the nucleus to upregulate *STIL* expression. Furthermore, HIF1α was reported to drive *FOXM1* expression [10, 11]. Thus, STIL can associate with FOXM1 to modulate the expression of downstream target genes involved in the EMT process and stemness, and consequently promotes tumor development, metastasis, and poor prognosis

Tumorigenesis is a complex and dynamic process consisting of three major stages: initiation, progression, and metastasis. In the present studies, we identify a novel STIL-mediated mechanism that promotes tumor progression and metastasis. Our collective in vitro and in vivo results on cell proliferation, colony formation, and xenograft tumor assay (Additional file 2: Fig. S2) support a role of STIL in tumor progression. Furthermore, we demonstrated that STIL is associated with FOXM1 (Fig. 4f), and that this association promotes tumor metastasis by activating FOXM1-regulated downstream genes (e.g., *SLUG*, *NANOG*, *SOX2*, *POU5F1*, *SKP2*, *CDC25A*, *CCNB1*, *CCNB2*, *CDK1*, *PLK1*, *AURKA*, *AURKB* and *BUBR1*) that are involved in the EMT, CS, and cell cycle (Figs. 3b, 4i, and Additional file 2: Fig. S7b). Importantly, we demonstrate that hypoxia is a new factor contributing to STIL upregulation in cancers. HIF1-α directly binds the *STIL* promoter under hypoxia (Fig. 6e), consequently potentiating hypoxia-induced tumor metastasis. A model showing how STIL contributes to tumor development via the FOXM1-mediated transcriptional activation under hypoxia is shown in Fig. 7.

Centrosome abnormalities are commonly observed in human cancers and are correlated with aneuploidy and poor patient prognosis. Previous studies used mouse models to focus on PLK4, which is a key regulator of centrosome duplication [112, 113]. However, the studies in mice with high expression of PLK4 provided contradictory results on the contribution of centrosome amplification to tumor progression. For example, centrosome amplification in neural progenitor cells resulted in microcephaly but did not promote tumorigenesis [114]. Furthermore, Kulukian et al. [115] and Vitre et al. [116] reported that overexpression of PLK4 in the skin epidermis induced an increase in centrosome number but failed to initiate or promote tumorigenesis in skin. In contrast, Sercin et al. [39] showed that PLK4 overexpression accelerates skin tumor formation in mice lacking P53 and Levine et al. [37] demonstrated that supernumerary centrosomes are sufficient to drive tumorigenesis in multiple tissues of mice. Thus, the issue of whether direct associations exist between centrosome abnormalities and cancers remains unclear.

In this study, we examined the T (tumor)/N (non-malignant) ratio of 14 centriolar/centrosomal genes in lung cancer patients and their paired adjacent non-malignant lung tissues. *STIL* showed the highest T/N ratio (3.5) among the studied genes (Table 1); this was found in patients with lung cancer, and was even higher than the T/N ratio (1.5) of PLK4 in these patients. Further analysis also showed that the *STIL* mRNA level was significantly increased in many other types of cancers (Additional file 1: Table S1a) and its high expression is associated with poor prognosis in patients with many cancer types (Additional file 1: Table S1b). STIL was previously reported to be present in the cytosol, specifically in the centriole [43], and act as a master regulator of PLK4 in initiating centriole duplication [45–49]. Here, we reveal an unexpected novel role of STIL in the nucleus. In addition to centriolar STIL, a subset of STIL translocate into the nucleus and function as the coactivator to enhance the downstream FOXM1-drievn genes via the association between STIL and FOXM1, and consequently contributes to the metastasis. This finding may explain the elevated STIL expression in lung cancer patients with metastatic lymph nodes or brain metastases.

A remaining open question is: Do the extra centrosomes induced by excess STIL promote tumor initiation and drive spontaneous tumorigenesis? The answer is not yet clear. Using si-SASS6 to block excess STIL-induced centriole amplification, we found that the migration and invasion abilities were significantly reduced in the si-SASS6-treated cells harboring excess STIL (Additional file 2: Fig. S4a). However, the si-SASS6 treatment does not completely block the migration/invasion abilities of STIL overexpressing cells (Additional file 2: Fig. S4a). These findings suggest that in addition to the excess STIL-mediated cancer cell migration and invasion, the possibility of supernumerary centrosome aberration-triggered tumorigenesis (e.g. aneuploidy and/or tissue architecture disruption) [20] can’t be ruled out. Future experiments are needed to clarify this discrepancy.

Finally, it has been proposed that the small increases in centrosome number induced by a low-to-moderate level of PLK4 are permissive for tumor development, whereas high levels of PLK4 trigger larger number of centrosomes and are likely to be harmful for long-term cell survival [37]. Since STIL is a master regulator of PLK4, we speculate that a low-to-moderate level of STIL could promote tumor initiation, as seen for PLK4, via a yet-unknown mechanism. Future experiments by generating a transgenic mouse line with low to moderate expression level of STIL could be a way to test the role of STIL in the initial stage of tumorigenesis.

In summary, we herein show that STIL is significantly up-regulated in lung and many other types of cancers, and that its expression level is highly correlated with patient survival, implicating its potential application in cancer detection and as a prognostic marker. Importantly, we demonstrate that STIL plays a versatile role in multistage tumorigenesis through the HIF1α-STIL-FOXM1 axis, and therefore may serve as a promising target for cancer therapy.

## Conclusion

Our findings indicate that the centriolar protein STIL functions not only as a key regulator in centriole duplication but also as a transcriptional coactivator that regulates EMT and stemness to promote tumor metastasis. Our findings show that a subset of STIL enter the nucleus, which interact with FOXM1 to activate its downstream target genes in metastasis. Furthermore, we provide evidence to show that HIF1α directly binds to *STIL* promoter and drives *STIL* gene expression under hypoxia. Thus, STIL can serve as a potential diagnostic marker for early lung cancer detection, and a promising therapeutic target for lung cancer treatment.

### Supplementary Information

**Additional file 1.** Additional Tables S1–S4.

**Additional file 2. **Additional Figures S1–S8.

## Data Availability

All data relevant to the study are included in the article and in additional files. The reagents used in this publication are available from the corresponding author on reasonable request.

## Abbreviations

ADC: Adenocarcinoma
ARG2: Arginase 2
AUC: Area under the curve
AURKA: Aurora kinase A
AURKB: Aurora kinase B
BUBR1/BUB1B: Budding uninhibited by benzimidazoles 1 homolog beta
CCK-8: Cell counting kit-8
CCNB1: Cyclin B1
CCNB2: Cyclin B2
CCND1: Cyclin D1
CDC25A: Cell Division Cycle 25A
CDK1: Cyclin dependent kinase 1
CEBPB: CCAAT/enhancer-binding protein beta
CEP63: Centrosomal protein 63
CEP120: Centrosomal protein 120
CEP152: Centrosomal protein 152
CEP135: Centrosomal protein 135
CEP295: Centrosomal protein 295
CETN1: Centrin 1
ChIP: Chromatin immunoprecipitation
CPAP: Centrosomal P4.1-associated protein
CS: Cancer stemness
DMEM: Dulbecco’s Modified Eagle’s Medium
Dox: Doxycycline
EMT: Epithelial–mesenchymal transition
FBS: Fetal bovine serum
FOXM1: Forkhead box protein M1
GEO: Gene Expression Omnibus
GSEA: Gene set enrichment analysis
HIF1α: Hypoxia-inducible factor 1α
ICC: Immunocytochemistry
IHC: Immunohistochemistry
IF: Immunofluorescent
IVIS: In vivo imaging system
MCPH: Autosomal recessive primary microcephaly
MT: Microtubule
MTOC: Microtubule organizing center
PCM: Pericentriolar material
PLK1: Polo like kinase 1
PLK4: Polo-like kinase 4
POC1B: Proteome of centriole protein 1 beta
POC5: Proteome of centriole protein 5
qPCR: Quantitative polymerase chain reaction
R: Pearson correlation coefficient
ROC: Receptor operating characteristics
RTTN: Rotatin
SAS6: Spindle assembly abnormal protein 6
SAT2: Spermidine/Spermine N1-acetyltransferase family member 2
SCC: Squamous cell carcinoma
SD: Standard derivation
SKP2: S-phase kinase associated protein 2
SNP: Single-nucleotide polymorphism
SPICE1: Spindle and centriole-associated protein 1
STIL: SCL/TAL1-interrupting locus
TCGA: The Cancer Genome Atlas
VEGF: Vascular endothelial growth factor
