# Correction: A novel HIF1α-STIL-FOXM1 axis regulates tumor metastasis

**DOI:** 10.1186/s12929-024-01021-w

**Published:** 2024-04-09

**Authors:** Yi-Wei Wang, Shu-Chuan Chen, De-Leung Gu, Yi-Chen Yeh, Jhih-Jie Tsai, Kuo-Tai Yang, Yuh-Shan Jou, Teh-Ying Chou, Tang K. Tang

**Affiliations:** 1 Institute of Biomedical Sciences, Academia Sinica, 128 Academia Rd., Sec. 2, Taipei, 11529 Taiwan; 2 Department of Pathology and Laboratory Medicine, Taipei Veterans General Hospital, Taipei, Taiwan; 3 Present Address: Dept. of Animal Science, National Pingtung University of Science and Technology, Pingtung, Taiwan

**Correction** **:** **J Biomed Sci 29, 24 (2022)**

**https://doi.org/10.1186/s12929-022-00807-0**

After publication of this article [1], it was brought to our attention that the figure 6, supplementary figure s5, Supplementary Figure S8 need to be corrected.

The Incorrect figure 6 is:


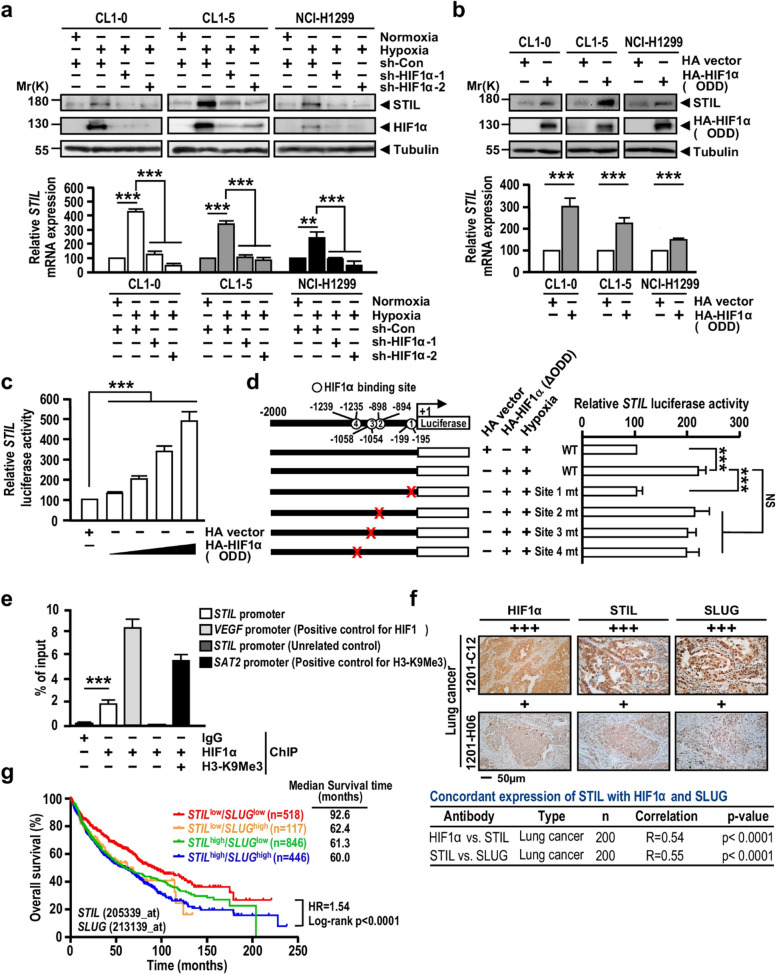


The correct figure 6 is:


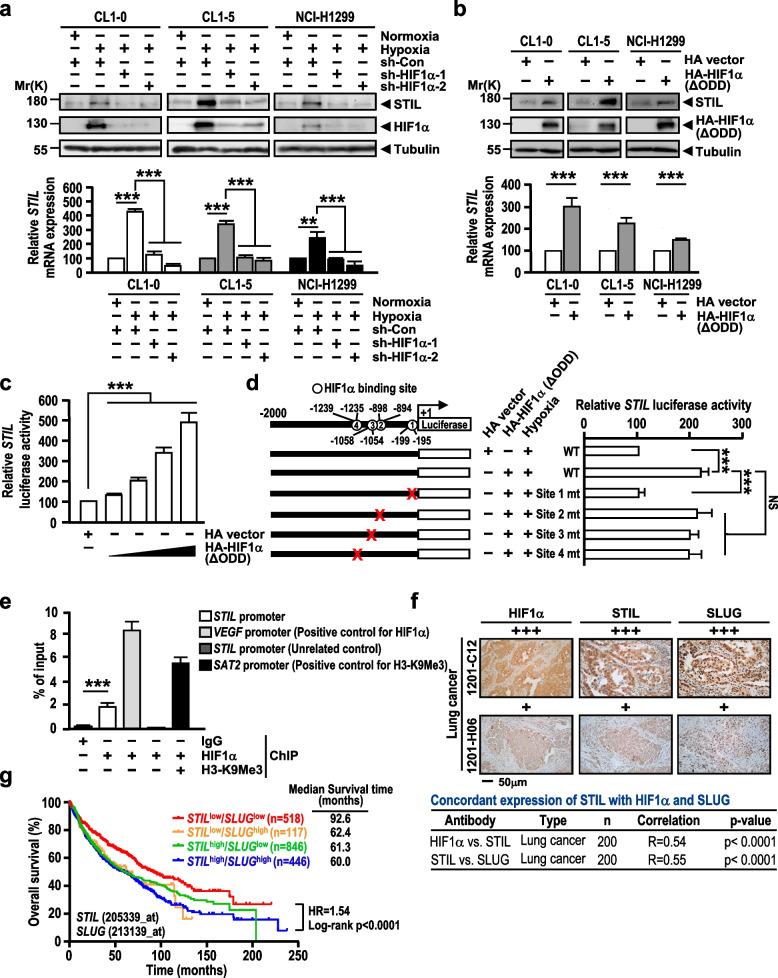


The Incorrect supplementary figure s5 is:


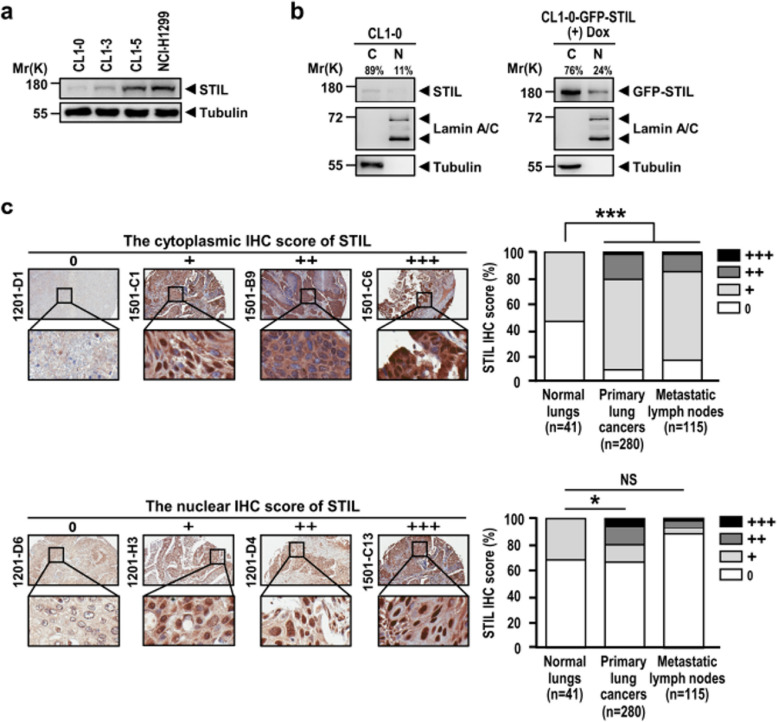


The correct supplementary figure s5 is:


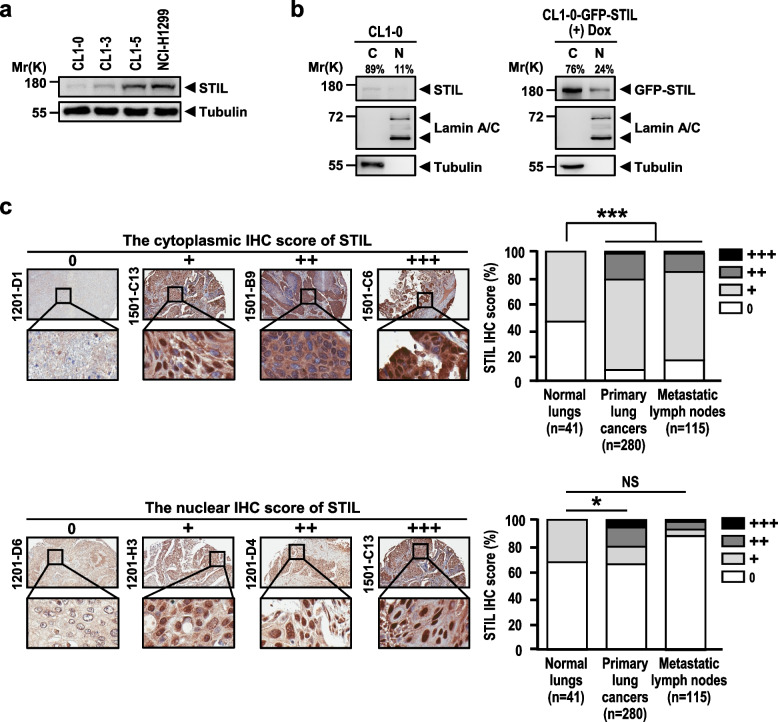


The Incorrect Supplementary Figure S8 is:


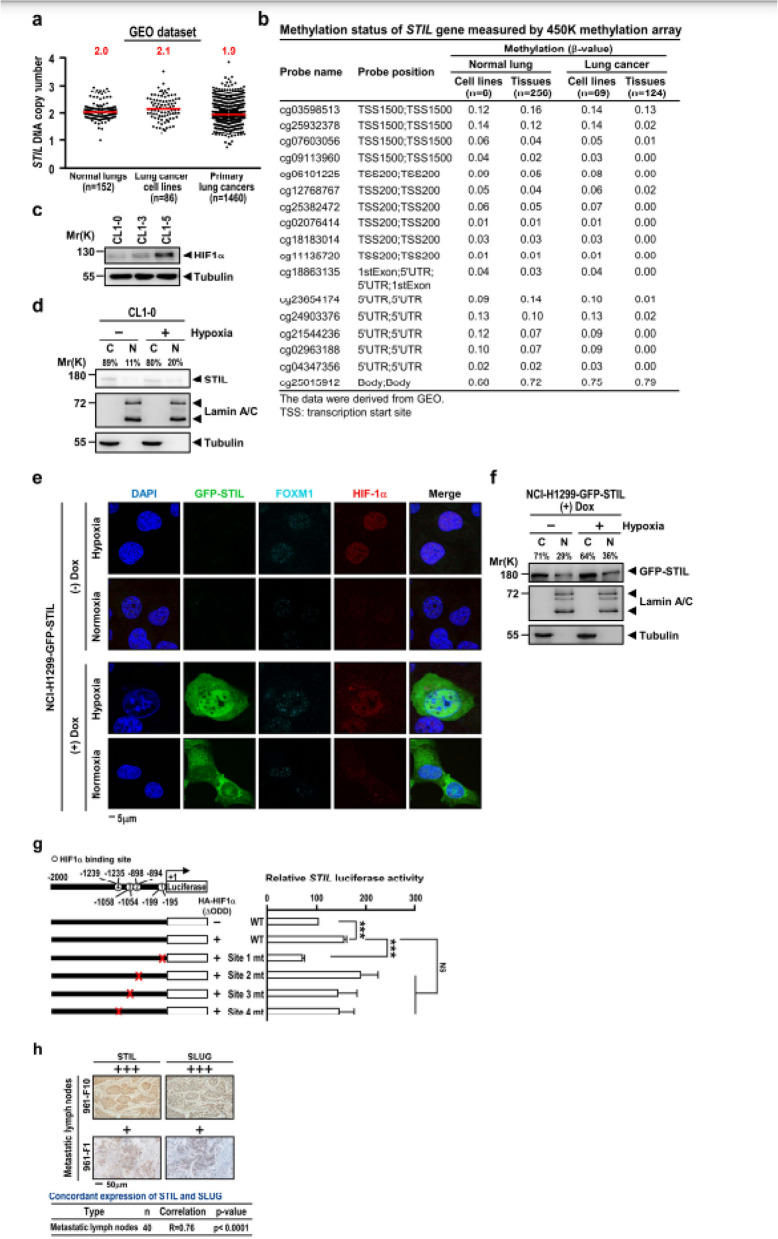


The correct Supplementary Figure S8 is:


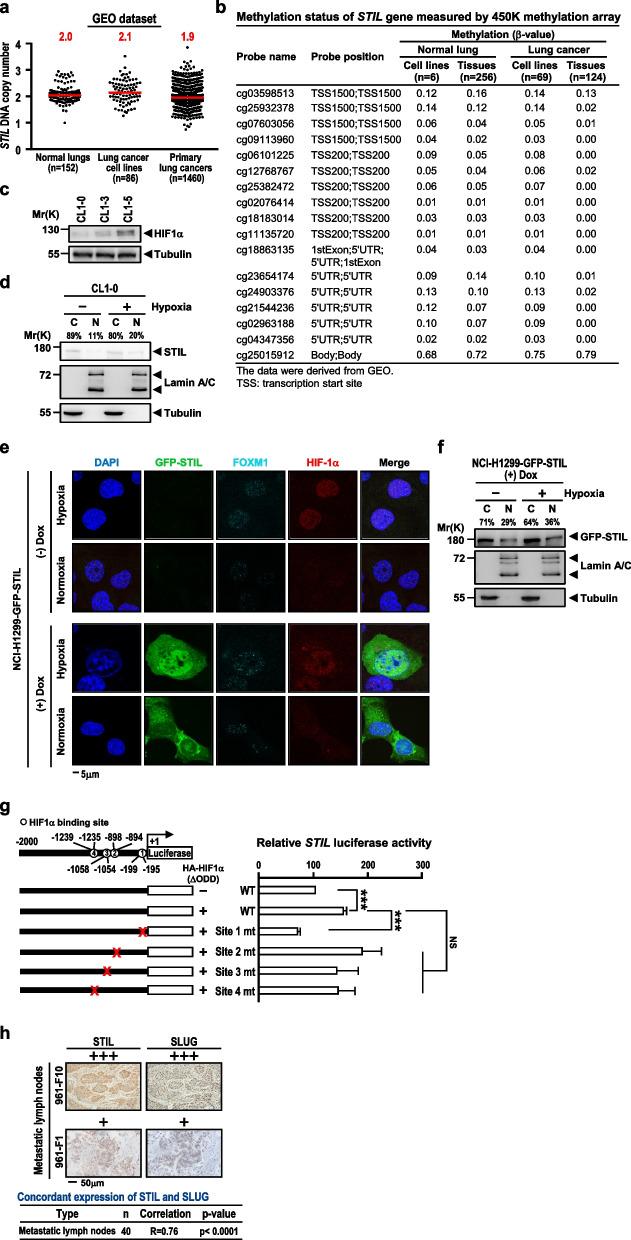


The original publication has been corrected.
