# Supplementary material for: A novel HIF1α-STIL-FOXM1 axis regulates tumor metastasis
Source: J Biomed Sci. 2022 Apr 1;29:24. doi: 10.1186/s12929-022-00807-0 (PMC8973879; doi:10.1186/s12929-022-00807-0)
Supplement: Supplementary file 1 — Additional file 1. Additional Tables S1–S4. [file 12929_2022_807_MOESM1_ESM.pdf]

Supplementary Material

Additional File 1 contains 4 supplementary tables

A novel HIF1α-STIL-FOXM1 axis regulates tumor metastasis

Yi-Wei Wang[1], Shu-Chuan Chen[1], De-Leung Gu[1], Yi-Chen Yeh[2], Jhih-Jie Tsai[1], Kuo-Tai Yang[1,#], Yuh-Shan Jou[1], Teh-Ying Chou[2], and Tang K. Tang[1, *]

[1]Institute of Biomedical Sciences, Academia Sinica, Taipei, Taiwan

[2]Department of Pathology and Laboratory Medicine, Taipei Veterans General Hospital, Taipei, Taiwan.

#Present address: Dept. of Animal Science, National Pingtung University of Science and Technology, Pingtung, Taiwan.

* Corresponding author: Dr. Tang K. Tang

E-mail: tktang@ibms.sinica.edu.tw

Supplementary Table S1

a

***STIL* expression in cancers**

Cancer type	***STIL* expression (RNA-seq)**			
	**Tumor**	**Non-malignant**	**Ratio (T/N)**	**p-value (T vs. N)**
Lung squamous cell carcinoma	8.6 (n=349)	5.0 (n=43)	1.8	2.9E-108
Breast invasive carcinoma	8.1 (n=917)	5.9 (n=107)	1.4	1.9E-83
Lung adenocarcinoma	7.8 (n=391)	5.1 (n=57)	1.5	4.1E-61
Prostate adenocarcinoma	7.5 (n=176)	5.6 (n=43)	1.3	1.2E-31
Uterine corpus endometrial earcinoma	8.4 (n=369)	5.3 (n=11)	1.6	4.8E-29
Kidney renal clear cell carcinima	6.2 (n=479)	5.3 (n=71)	1.2	2.9E-25
Head and neck squamous cell carcinoma	8.7 (n=302)	7.6 (n=37)	1.2	4.4E-23
Liver hepatocellular carcinoma	6.8 (n=69)	4.3 (n=35)	1.6	5.0E-22
Bladder urothelial Carcinoma	8.6 (n=150)	5.1 (n=16)	1.7	2.0E-20
Kidney renal papillary cell carcinoma	6.4 (n=75)	4.5 (n=25)	1.4	1.9E-17
Colon adenocarcinoma	8.9 (n=192)	7.5 (n=18)	1.2	6.9E-16
Kidney chromophobe	5.5 (n=65)	4.5 (n=25)	1.2	1.0E-07
Thyroid carcinoma	5.6 (n=485)	5.3 (n=58)	1.1	2.6E-04

All data are derived from TCGA datasets.
Ranking is based on the p-value.
Significance is determined by t-test.

b

Kaplan-Meier analysis of overall survival

Cancer type	**Median survival (months)**			
	**STIL^{high}**	**STIL^{low}**	**p-value**	**HR**
Lung squamous cell carcinoma	44.5 (n=231)	64.4 (n=264)	0.042	1.32
Lung adenocarcinoma	42.9 (n=283)	54.1 (n=221)	0.005	1.53
Uterine corpus endometrial carcinoma	44.4 (n=341)	111.6 (n=201)	0.002	2.04
Kidney renal clear cell carcinima	23.6 (n=200)	54.1 (n=300)	<0.0001	2.15
Head and neck squamous cell carcinoma	58.7 (n=134)	47.6 (n=365)	0.015	0.66
Liver hepatocellular carcinoma	25.5 (n=99)	71.0 (n=271)	<0.0001	2.12
Kidney renal papillary cell carcinoma	23.2 (n=73)	87.4 (n=214)	<0.0001	4.33
Esophageal adenocarcinoma	13.6 (n=25)	46.7 (n=55)	0.003	2.64
Esophageal squamous cell carcinoma	48.6 (n=37)	21.6 (n=44)	0.007	0.27
Ovarian cacner	41.6 (n=122)	48.0 (n=251)	0.007	0.27
Pancreatic ductal adenocarcinoma	10.2 (n=123)	19.7 (n=54)	<0.0001	2.79
Rectum adenocarcinoma	52.2 (n=122)	36.5 (n=43)	0.008	0.37
Sarcoma	22.9 (n=182)	41.1 (n=77)	0.006	1.95
Stomach adenocarcinoma	46.9 (n=186)	25.9 (n=185)	0.005	0.63

All data are derived from Kaplan-Meier Plotter dataset (RNA-seq).
HR: Hazard Ratio
Significance is determined by the log-rank test.

Supplementary Table S2

Lung cancer subtypes used in Table 1

Subtype	Case (n)	Percentage (%)
Adenocarcinoma	163	100
Squamous cell carcinoma	0	0
Others	0	0
Total	163	

Lung cancer subtypes used in Fig 1B

Subtype	Case (n)	Percentage (%)
Adenocarcinoma	392	53
Squamous cell carcinima	348	47
Others	0	0
Total	740	

Lung cancer subtypes used in Fig 1C

Subtype	Case (n)	Percentage (%)
Adenocarcinoma	78	27.8
Squamous cell carcinima	129	46.0
Others	73	26.2
Total	280	

Lung cancer subtypes used in Fig 1D

Subtype	Case (n)	Percentage (%)
Adenocarcinoma	955	100
Squamous cell carcinima	0	0
Others	0	0
Total	955	

Lung cancer subtypes used in Fig 1E

Subtype	Case (n)	Percentage (%)
Adenocarcinoma	865	45.0
Squamous cell carcinima	675	35.0
Others	386	20.0
Total	1926	

Lung cancer subtypes used in Supplementary Fig S1

Subtype	Case (n)	Percentage (%)
Adenocarcinoma	504	50
Squamous cell carcinima	495	50
Others	0	0
Total	999	

Supplementary Table S3

Antibody information

Antibody	Assay	Company	Cat#	Dilution
Anti-E-cadherin	WB	BD Bioscience	610181	1/500
Anti-EGFR	WB	Cell signaling	4267	1/1000
Anti-Flag	WB	Sigma	F3165	1/1000
Anti-FOXM1	WB	Santa Cruz	SC-376471	1/500
Anti-GFP	WB	Takara	632381	1/2000
Anti-HA	WB	Covance	MMS-101P	1/1000
Anti-HIF1α	WB	BD Bioscience	610958	1/1000
Anti-Histone H3	WB	Cell signaling	4499	1/1000
Anti-Lamin A/C	WB	Santa Cruz	SC-376248	1/1000
Anti-N-cadherin	WB	Abcam	AB18203	1/500
Anti-SAS6	WB	Abnova	H00163786-B01P	1/1000
Anti-SLUG	WB	Abcam	AB27568	1/500
Anti-STIL	WB	Bethyl	A302-442A	1/1000
Anti-Tubulin (DM1A)	WB	Sigma	T6199	1/30000
Anti-Vimentin	WB	Santa Cruz	SC-6260	1/3000
Anti-FOXM1	ChIP	GeneTex	GTX102170	1/200
Anti-HIF1α	ChIP	Abcam	AB2185	1/200
Anti-Histone H3 (tri-methyl K9)	ChIP	Abcam	AB8898	1/200
Anti-STIL	ChIP	Sigma	HPA046543	1/100
Anti-HIF1α	IHC	Abcam	AB2185	1/500
Anti-SLUG	IHC	Abcam	AB85936	1/500
Anti-STIL	IHC	Sigma	HPA046543	1/200
Anti-Arp3	IF	Abcam	AB49671	1/200
Anti-Centrin-3	IF	Abnova	H00001070-M01	1/400
Anti-FOXM1	IF	Santa Cruz	SC-376471	1/100
Anti-HIF1α	IF	Abcam	AB2185	1/100

Supplementary Table S4

Sequences of the oligonucleotides for qPCR, ChIP, sh-RNA, and si-RNA

Name	Assay	Sequence
SLUG	qPCR	Forward: ATGCATATTCGGACCCACACATTAC<br>Reverse: AGATTTGACCTGTCTGCAAATGCTC
STIL	qPCR	Forward: CCCCACTGCCATCTTACTGT<br>Reverse: ATTGCTGTGGGGAGAACAAC
SNAI1 (SNAIL)	qPCR	Forward: CCTCCCTGTCAGATGAGGAC<br>Reverse: CCAGGCTGAGGTATTCCTTG
HIF1 α	qPCR	Forward: GAAAGCGCAAGTCTTCAAAG<br>Reverse: TGGGTAGGAGATGGAGATGC
TWIST	qPCR	Forward: GCCGGAGACCTAGATGTCATTG<br>Reverse: CACGCCCTGTTTCTTTGAATTT
MMP9	qPCR	Forward: TTGACAGCGACAAGAAGTGG<br>Reverse: GCCATTCACGTCGTCCTTAT
CD44	qPCR	Forward: CGGACACCATGGACAAGTTT<br>Reverse: GAAAGCCTTGCAGAGGTCAG
NANOG	qPCR	Forward: ATGCCTCACACGGAGACTGT<br>Reverse: AAGTGGGTTGTTTGCCTTTG
SOX2	qPCR	Forward: TGATGGAGACGGAGCTGAA<br>Reverse: GGGCTGTTTTTCTGGTTGC
POU5F1 (OCT4)	qPCR	Forward: TCGAGAACCGAGTGAGAGG<br>Reverse: GAACCACACTCGGACCACA
β*-actin*	qPCR	Forward: GCTGTGCTACGTCGCCCTG<br>Reverse: GGAGGAGCTGGAAGCAGCC
SKP2	qPCR	Forward: TACAGAAAGAATCTCCAGAAATCAGATC<br>Reverse: GGAAAAATTCCTGAAAGCAGTCA
CDC25A	qPCR	Forward: TTCCTCTTTTTACACCCCAGTCA<br>Reverse: TCGGTTGTCAAGGTTTGTAGTTC
CCNB1	qPCR	Forward: TTTCGCCTGAGCCTATTTTG<br>Reverse: GCACATCCAGATGTTTCCATT
CCNB2	qPCR	Forward: GCGTTGGCATTATGGATCG<br>Reverse: TCTTCCGGGAAACTGGCTG
CDK1	qPCR	Forward: TTTTCAGAGCTTTGGGCACT<br>Reverse: CCATTTTGCCAGAAATTCGT
PLK1	qPCR	Forward: AACGACTTCGTGTTCGTGGT<br>Reverse: AGGGCTTTCCTCCTCTTGTG
AURKA	qPCR	Forward: CCACCTTCGGCATCCTAATA<br>Reverse: TCCAAGTGGTGCATATTCCA
AURKB	qPCR	Forward: GATGGCCCAGAAGGAGAACT<br>Reverse: AGGCTCTTTCCGGAGGACT
BUBR1	qPCR	Forward: TCGTGGCAATACAGCTTCAC<br>Reverse: GGTCAATAGCTCGGCTTCC
CCND1	qPCR	Forward: CCTGTCCTACTACCGCCTCA<br>Reverse: CAGTCCGGGTCACACTTGA
CEBPB	qPCR	Forward: AACTCTCTGCTTCTCCCTCTG<br>Reverse: AAGCCCGTAGGAACATCTTT
SLUG promoter	ChIP	Forward: CACACGGGCATACGTGTTAC<br>Reverse: TCGCCATGGCTATTATTTCC
SLUG promoter (unrelated control)	ChIP	Forward: GAGCTTCCTGGCTCACAGAC<br>Reverse: GAATGCTTGTGTCCCCCTAA
STIL promoter	ChIP	Forward: ACCGTTGACGAAATGAAAGG<br>Reverse: GGTCTGTTTGCAGGGTAGGA
STIL promoter (unrelated control)	ChIP	Forward: GATCCCATCCACGGTATCAC<br>Reverse: TCTGTGGTCTTCCTCCCATT
SAT2 promoter<br>(Positive control for H3-K9M3)	ChIP	Forward: CTGCAATCATCCAATGGTCG<br>Reverse: GATTCCATTCGGGTCCATTC
ARG2 promoter<br>(Positive control for FOXM1)	ChIP	Forward: TTGACAGGAGCAGGGAAGTATTGTAGA<br>Reverse: CATTTGATTTGCCTGAAGGCTGATTTGT
VEGF promoter<br>(Positive control for HIF1α)	ChIP	Forward: CAGGAACAAGGGCCTCTGTCT<br>Reverse: TGTCCCTCTGACAATGTGCCATC

Supplementary Table S4-continued

Sequences of the oligonucleotides for qPCR, ChIP, sh-RNA, and si-RNA

Name	Assay	Sequence
sh-STIL-1	shRNA	GATCAGTTATTCCTACAAGTT
sh-STIL-2	shRNA	GTCTGGAATTACACATATCTA
sh-FOXM1-1	shRNA	AGGACCACTTTCCCTACTTTA
sh-FOXM1-2	shRNA	GCCAATCGTTCTCTGACAGAA
sh-HIF1α-1	shRNA	GTGATGAAAGAATTACCGAAT
sh-HIF1α-2	shRNA	TGCTCTTTGTGGTTGGATCTA
si-Con	siRNA	UGGUUUACAUGUCGACUAA<br>UGGUUUACAUGUUGUGUGA<br>UGGUUUACAUGUUUUCUGA<br>UGGUUUACAUGUUUUCCUA
si-SASS6	siRNA	GGACAGUCUCUUCGAAUUA<br>UGUUGAAUGCCACGAGAAA<br>CAACUGGACUUUACACGAA<br>GAGAGUAAGUAUAAGAAUG
