# Supplementary material for: A novel HIF1α-STIL-FOXM1 axis regulates tumor metastasis
Source: J Biomed Sci. 2022 Apr 1;29:24. doi: 10.1186/s12929-022-00807-0 (PMC8973879; doi:10.1186/s12929-022-00807-0)
Supplement: Supplementary file 2 — Additional file 2. Additional Figures S1–S8. [file 12929_2022_807_MOESM2_ESM.pdf]

Supplementary Material

Additional File 2 contains 8 supplementary figures

A novel HIF1α-STIL-FOXM1 axis regulates tumor metastasis

Yi-Wei Wang[1], Shu-Chuan Chen[1], De-Leung Gu[1], Yi-Chen Yeh[2], Jhih-Jie Tsai[1], Kuo-Tai Yang[1,#], Yuh-Shan Jou[1], Teh-Ying Chou[2], and Tang K. Tang[1,*]

[1]Institute of Biomedical Sciences, Academia Sinica, Taipei, Taiwan

[2]Department of Pathology and Laboratory Medicine, Taipei Veterans General Hospital, Taipei, Taiwan.

#Present address: Dept. of Animal Science, National Pingtung University of Science and Technology, Pingtung, Taiwan.

* Corresponding author: Dr. Tang K. Tang

E-mail: tktang@ibms.sinica.edu.tw

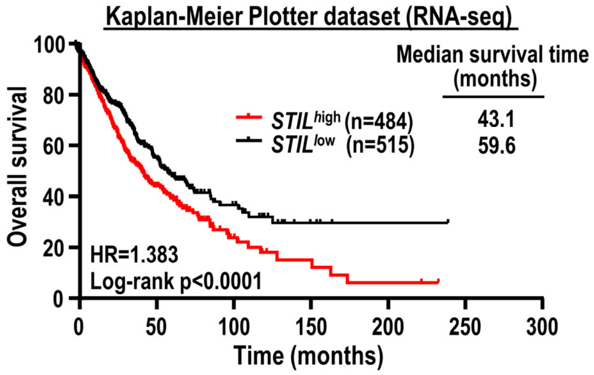

Supplementary Fig S1. *STIL* expression level is correlated with poor prognosis in patients with lung cancer. Kaplan-Meier survival analysis of *STIL* gene expression was performed using RNA-seq data of 999 lung cancers derived from Kaplan-Meier Plotter. The histologic subtypes of lung cancer patients are described in Additional file 1: Table S2. The median survival of the two groups is shown. Significance is determined by the log-rank test (p< 0.0001).

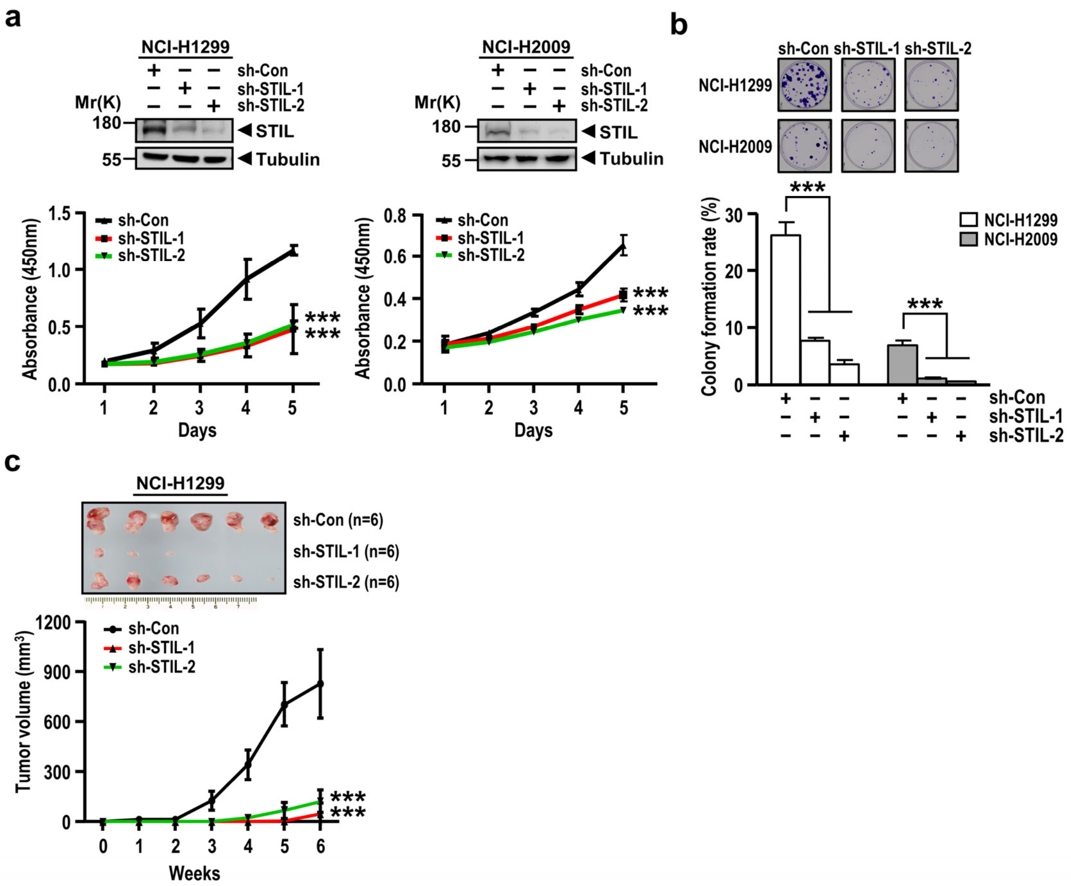

Supplementary Fig S2. STIL promotes oncogenic transformation. a Western blotting analysis of STIL protein expression in control (sh-Con) and STIL-knockdown (sh-STIL-1/sh-STIL-2) NCI-H1299 and NCI-H2009 cells (upper panel). Tubulin used as loading control. The cell proliferation rate was measured by a CCK-8 assay in control and STIL-knockdown cells (n = 3 independent experiments) (lower panel). **b** Colony numbers were determined by colony formation assay in control and STIL-knockdown NCI-H1299 and NCI-H2009 cells. Data represent the mean ± SD (n = 3 independent experiments). Significance is determined by t-test (***p< 0.001). **c** *In vivo* tumor formation was assessed by subcutaneous injection of STIL-knockdown NCI-H1299 cells into c-nude mice (n = 6 per group). Phase contrast images of tumor (upper panel) and tumor growth rate (lower panel) are shown.

Data information: In **a** and **c**, the significance is determined by two-way ANOVA (***p< 0.001).

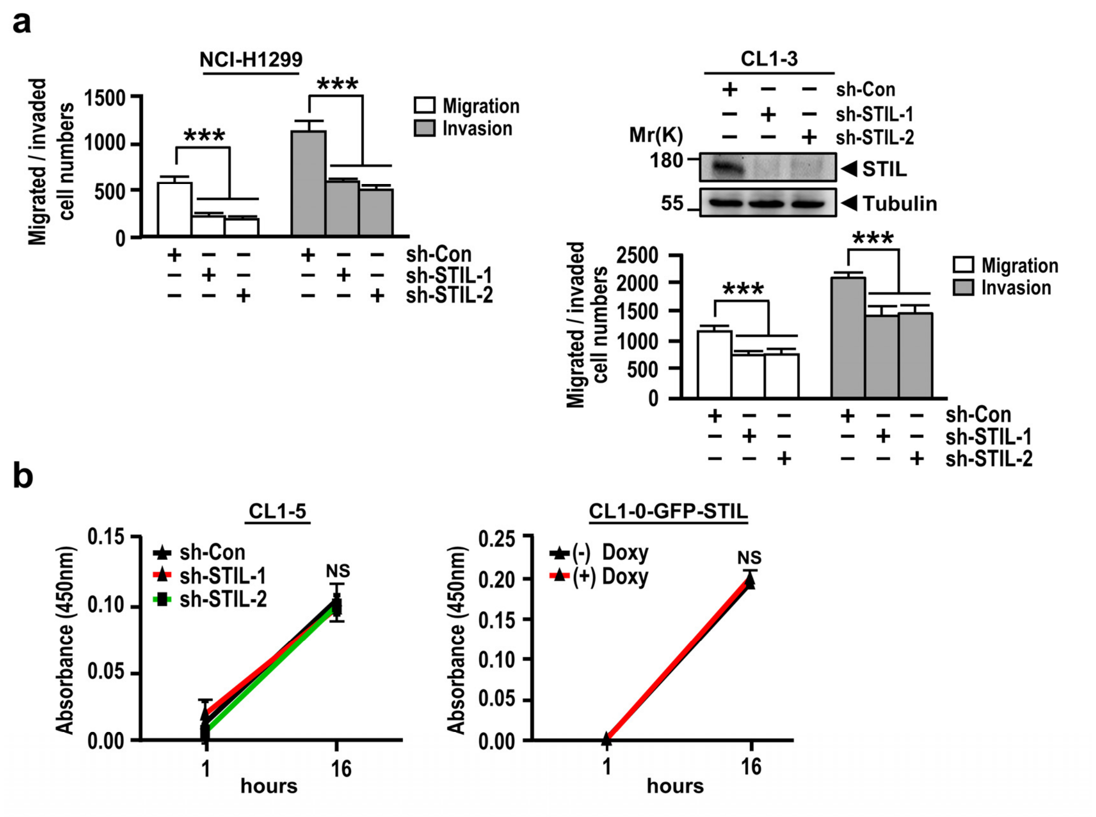

Supplementary Fig S3. Depletion of STIL impairs cell migration and invasion abilities in NCI-H1299 and CL1-3 cells. a Western blotting analysis of STIL protein levels in control (sh-Con) and STIL-knockdown (sh-STIL-1/sh-STIL-2) NCI-H1299 (Additional file 2: Fig S2a) and CL1-3 cells (right-upper panel). Tubulin used as loading control of Western blotting. Cell migration and invasion abilities were analyzed by Boyden chamber assay in STIL-knockdown NCI-H1299 and CL1-3 cells (lower panel). **b** Cell proliferation rate was measured after a 16-hour incubation time (a time period used for monitoring cell migration and invasion) by a CCK-8 assay in STIL-knockdown CL1-5 cells (left panel) and in CL1-0 cells overexpressing GFP-STIL under Dox treatment for 48 hrs (right panel).

Data information: Statistical data represent the mean ± SD (n = 3 independent experiments). Significance is determined by t-test (NS, not significant; *** $p<0.001$).

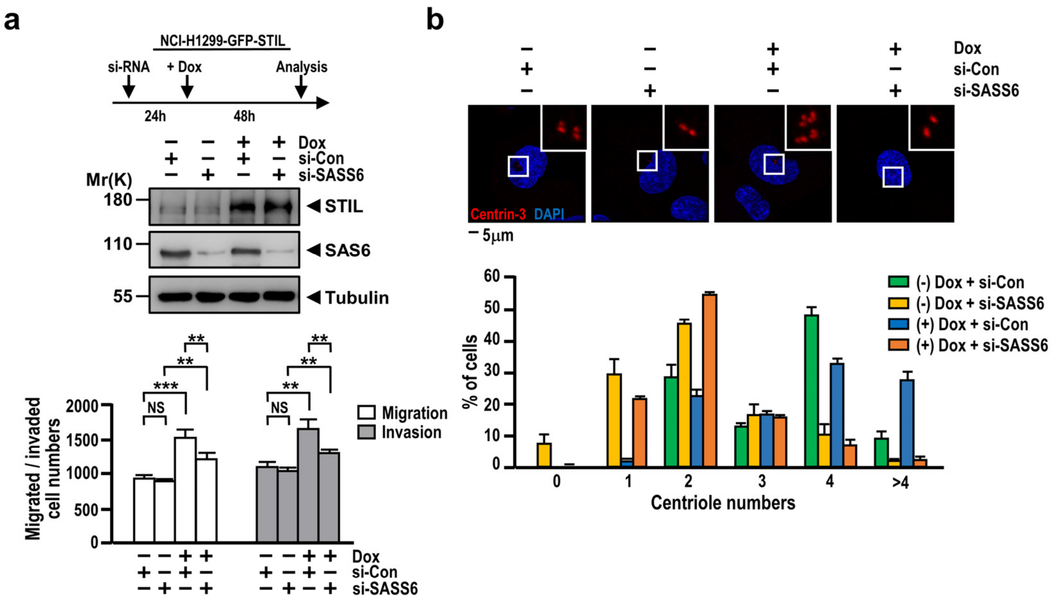

Supplementary Fig S4. In addition to centriole amplification, other STIL-involved mechanisms contribute to STIL-induced migration and invasion. a-b Analysis of STIL and SAS6 protein levels (a, upper panel), the migration/invasion abilities (a, lower panel), and the centriole numbers (b) in Dox inducible NCI-H1299 cells overexpressing GFP-STIL treated with si-Control (si-Con) or si-SASS6. Tubulin used as loading control of Western blotting analysis. Cell migration and invasion abilities of these cells were analyzed by Boyden chamber assay. Centriole numbers in treated cells were analyzed by centrin-3 staining. The confocal images (b, upper panel) and the percentages of cells with various centriole numbers in control and treated cells were shown (b, lower panel).

Data information: Statistical data represent the mean ± SD (n = 3 independent experiments). Significance is determined by t-test (NS, not significant; **p< 0.01; *** p<0.001).

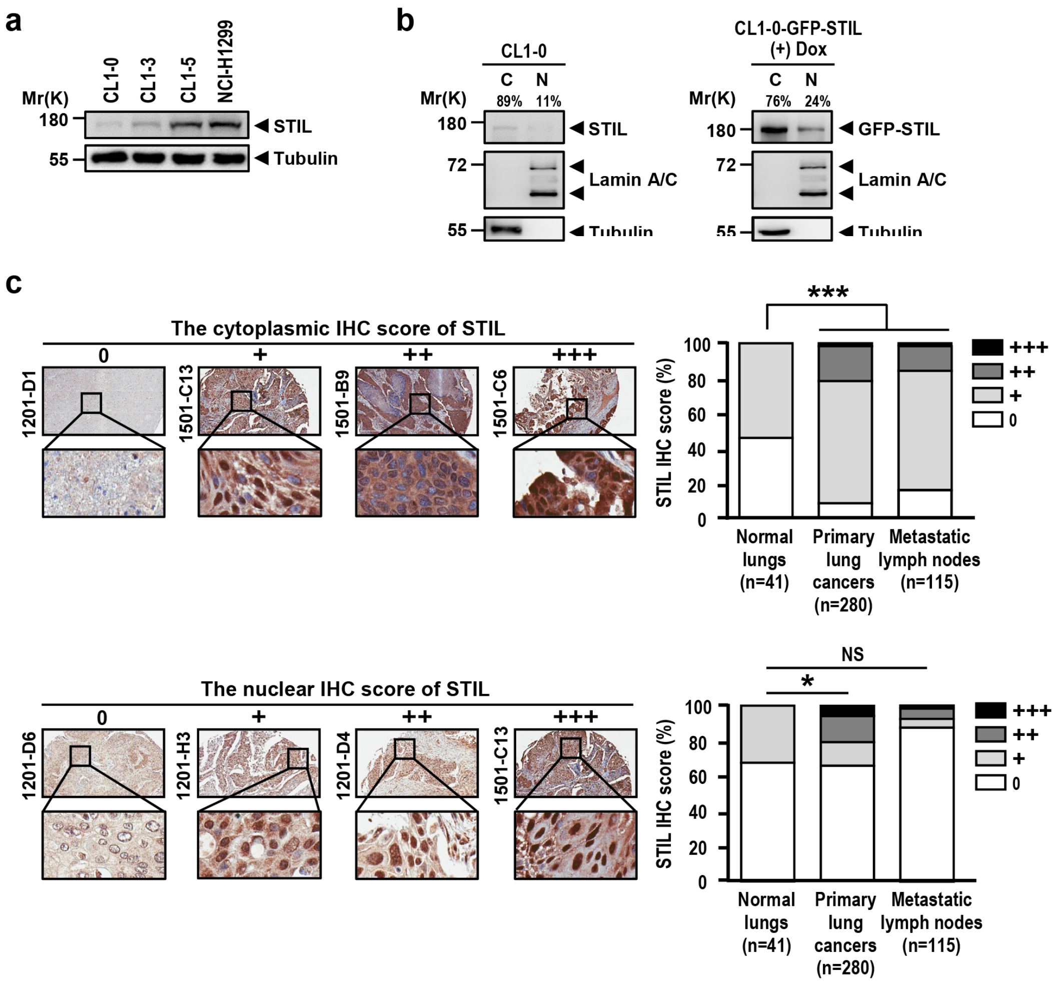

Supplementary Fig S5. A portion of STIL was detected in the nucleus. a Western blotting analysis of STIL protein levels in CL1-0, CL1-3, CL1-5, and NCI-H1299 cells. Tubulin used as loading control of Western blotting analysis. **b** STIL protein levels in cytoplasmic (C) and nuclear (N) fractions analyzed by Western blotting in CL1-0 cells (left panel) and CL1-0 cells overexpressing GFP-STIL under Dox treatment for 48 hours (right panel). Lamin A/C and tubulin were used as nuclear and cytoplasmic markers, respectively. The percentage of subcellular distribution is also shown. **c** The nuclear and cytoplasmic STIL protein levels were analyzed by IHC analysis in 41 normal lung tissue samples, 280 primary lung cancers, and 115 paired metastatic lymph nodes (derived from the pool of 280 primary lung cancers). Representative IHC images of STIL staining are shown (left panel). IHC staining intensity was grouped as 0 (negative), + (weak), ++ (moderate), and +++ (strong), and the percentage distribution is also included (right panel). Significance is determined by t-test (NS, not significant; *p< 0.05; ***p<0.001).

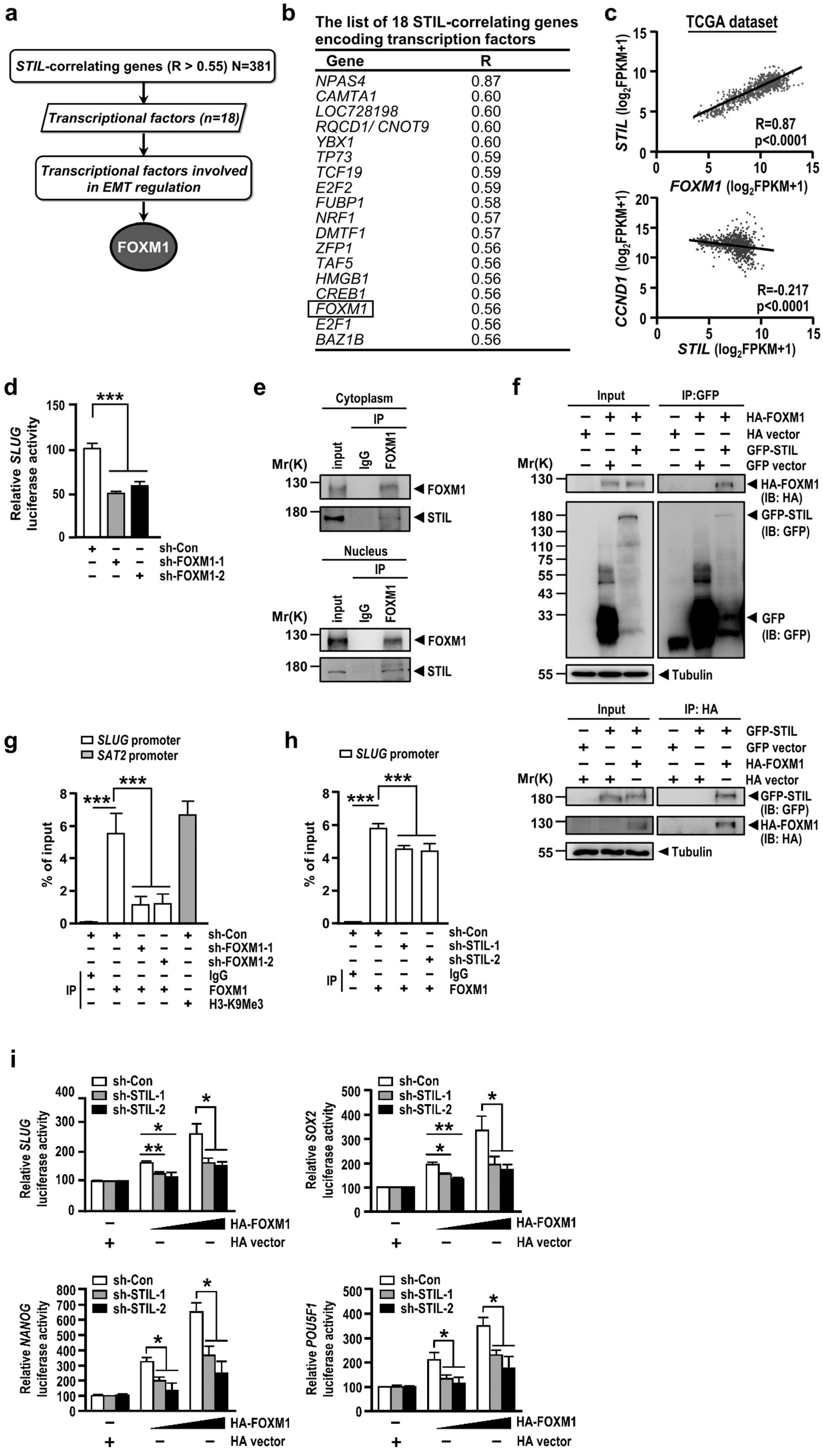

a
STIL-correlating genes (R > 0.55) N=381
Transcriptional factors (n=18)
Transcriptional factors involved in EMT regulation
FOXM1
b
The list of 18 STIL-correlating genes encoding transcription factors
Gene R
NPAS4 0.87
CAMTA1 0.60
LOC728198 0.60
RQCD1/ CNOT9 0.60
YBX1 0.60
TP73 0.59
TCF19 0.59
E2F2 0.59
FUBP1 0.58
NRF1 0.57
DMTF1 0.57
ZFP1 0.56
TAF5 0.56
HMGB1 0.56
CREB1 0.56
FOXM1 0.56
E2F1 0.56
BAZ1B 0.56
c
TCGA dataset
STIL (log2FPKM+1)
FOXM1 (log2FPKM+1)
R=0.87
p<0.0001
CCND1 (log2FPKM+1)
STIL (log2FPKM+1)
R=-0.217
p<0.0001
d
Relative SLUG luciferase activity
sh-Con
sh-FOXM1-1
sh-FOXM1-2
e
Cytoplasm
Nucleus
IP
input IgG FOXM1
Mr(K)
FOXM1
STIL
f
Input
IP:GFP
HA-FOXM1
HA vector
GFP-STIL
GFP vector
HA-FOXM1 (IB: HA)
GFP-STIL (IB: GFP)
GFP (IB: GFP)
Tubulin
Input
IP: HA
GFP-STIL
GFP vector
HA-FOXM1
HA vector
GFP-STIL (IB: GFP)
HA-FOXM1 (IB: HA)
Tubulin
g
SLUG promoter
SAT2 promoter
% of input
sh-Con
sh-FOXM1-1
sh-FOXM1-2
IgG
IP
FOXM1
H3-K9Me3
h
SLUG promoter
% of input
sh-Con
sh-STIL-1
sh-STIL-2
IgG
IP
FOXM1
i
sh-Con
sh-STIL-1
sh-STIL-2
Relative SLUG luciferase activity
Relative SOX2 luciferase activity
Relative NANOG luciferase activity
Relative POU5F1 luciferase activity
HA-FOXM1
HA vector

Supplementary Fig S6. Identification of potential STIL-correlating genes and a correlation between STIL and FOXM1. a Flow chart showing the strategy used to search for STIL-correlating genes. Microarray data of lung cancer cell lines were derived from EBI (E-MTAB-37). **b** The list of 18 *STIL*-correlating genes that encode transcriptional factors and show a Pearson's correlation coefficient (R)>0.55. **c** The concordant expression of *STIL* and *FOXM1* (upper panel) or *CCND1* (lower panel) was examined using the RNA-seq data of 100 non-malignant lung tissues and 740 lung cancer specimens derived from TCGA dataset. R is shown as 0.87 and -0.217, respectively. **d** The *SLUG* promoter-driven luciferase activity was determined by reporter assay in FOXM1-knockdown CL1-5 cells. **e** The association of endogenous STIL and FOXM1 in the cytoplasm and nucleus of CL1-5 cells was analyzed by co-IP and Western blotting using the indicated antibodies. **f** HEK293T cells were transiently co-transfected with GFP-STIL, HA-FOXM1, GFP vector, or HA vector constructs as indicated. Protein complexes were immunoprecipitated using anti-GFP antibody (upper panel) or anti-HA antibody (lower panel) and analyzed by Western blotting using the indicated antibodies. **g-h** The binding of FOXM1 to the *SLUG* promoter was analyzed by ChIP-qPCR assay in FOXM1-knockdown CL1-5 cells (g) or STIL-knockdown CL1-5 cells (h). Methylation of histone H3 (H3-K9Me3) on the *SAT2* gene was used as a positive control and IgG was used as a negative control for ChIP. **i** The *SLUG*, *NANOG*, *SOX2,* and *POU5F1* promoter activities were measured by reporter assay in STIL-knockdown CL1-5 cells transiently co-transfected with HA-FOXM1 and the indicated promoter-luciferase constructs.

Data information: In **d, and g-i,** data represent the mean ± SD (n = 3 independent experiments). Significance is determined by t-test (*p< 0.05; **p<0.01; *** p<0.001).

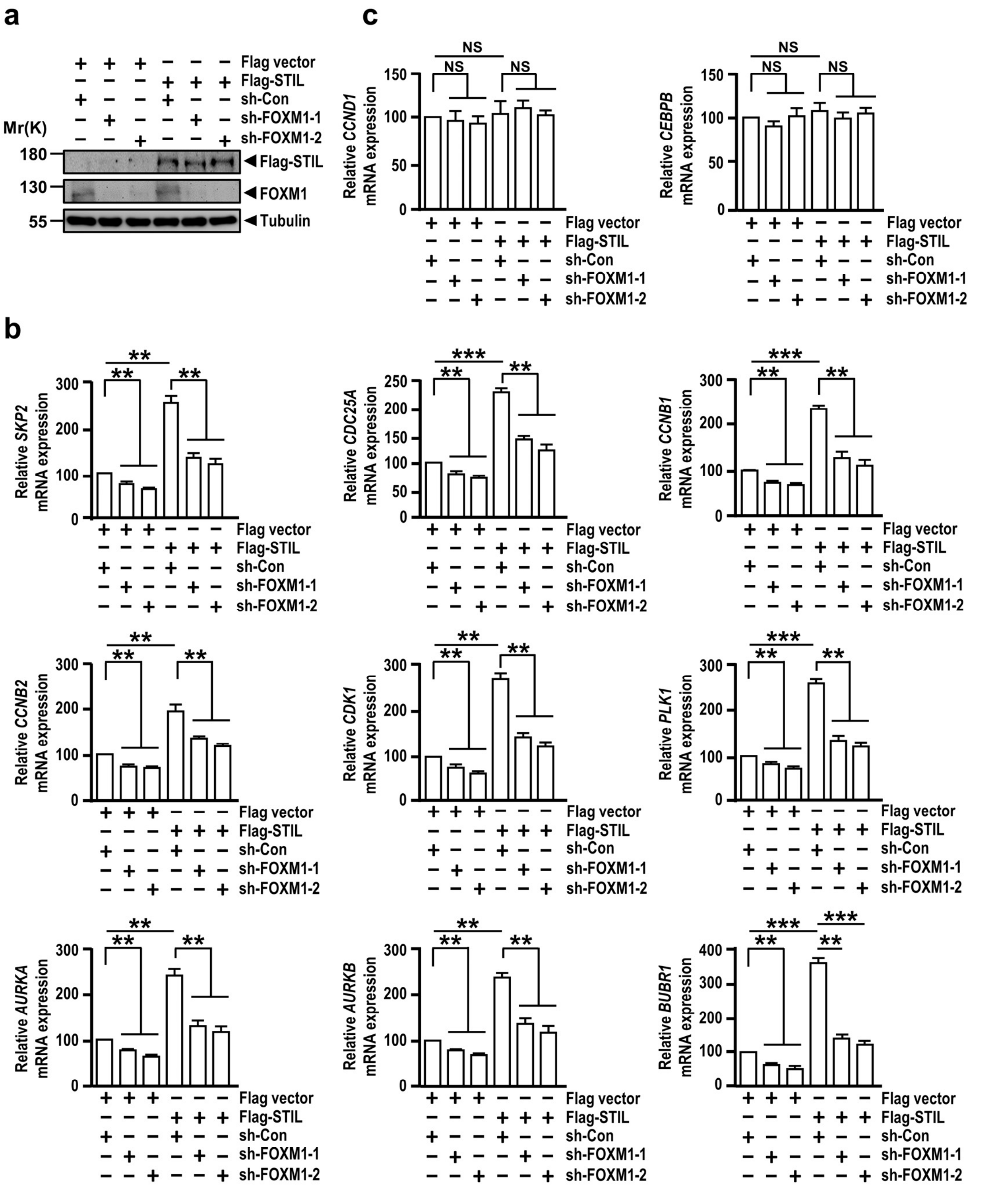

Supplementary Fig S7. The association of STIL with FOXM1 could upregulate some FOXM1-modulated genes involved in cell cycle. a The protein levels of Flag-STIL and FOXM1 were analyzed by Western blotting analysis in FOXM1-knockdown CL1-5 cells overexpressing Flag-STIL or Flag vector. Tubulin used as loading control of Western blotting. **b-c** The mRNA levels of *SKP2*, *CDC25A*, *CCNB1*, *CCNB2*, *CDK1*, *PLK1*, *AURKA*, *AURKB*, and *BUBR1* (b), *CCND1* and *CEBPB* (c) were measured by qPCR method in FOXM1-knockdown CL1-5 cells overexpressing Flag-STIL or Flag vector.

Data information: In **b-c,** data represent the mean ± SD (n = 3 independent experiments). Significance is determined by t-test (NS, not significant; **p< 0.01; *** p<0.001).

a

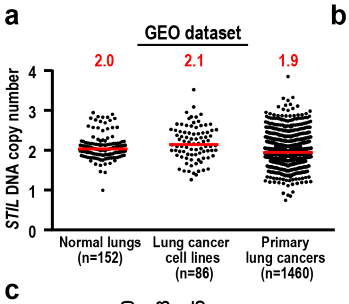

b

Methylation status of *STIL* gene measured by 450K methylation array

Probe name	Probe position	Methylation (β-value) Normal lung Cell lines (n=6)	Normal lung Tissues (n=256)	Lung cancer Cell lines (n=69)	Lung cancer Tissues (n=124)
cg03598513	TSS1500;TSS1500	0.12	0.16	0.14	0.13
cg25932378	TSS1500;TSS1500	0.14	0.12	0.14	0.02
cg07603056	TSS1500;TSS1500	0.06	0.04	0.05	0.01
cg09113960	TSS1500;TSS1500	0.04	0.02	0.03	0.00
cg06101225	TSS200;TSS200	0.09	0.05	0.08	0.00
cg12768767	TSS200;TSS200	0.05	0.04	0.06	0.02
cg25382472	TSS200;TSS200	0.06	0.05	0.07	0.00
cg02076414	TSS200;TSS200	0.01	0.01	0.01	0.00
cg18183014	TSS200;TSS200	0.03	0.03	0.03	0.00
cg11135720	TSS200;TSS200	0.01	0.01	0.01	0.00
cg18863135	1stExon;5'UTR; 5'UTR;1stExon	0.04	0.03	0.04	0.00
cg23654174	5'UTR;5'UTR	0.09	0.14	0.10	0.01
cg24903376	5'UTR;5'UTR	0.13	0.10	0.13	0.02
cg21544236	5'UTR;5'UTR	0.12	0.07	0.09	0.00
cg02963188	5'UTR;5'UTR	0.10	0.07	0.09	0.00
cg04347356	5'UTR;5'UTR	0.02	0.02	0.03	0.00
cg25015912	Body;Body	0.68	0.72	0.75	0.79

The data were derived from GEO.
TSS: transcription start site

c

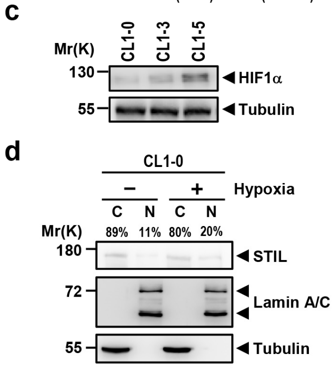

e

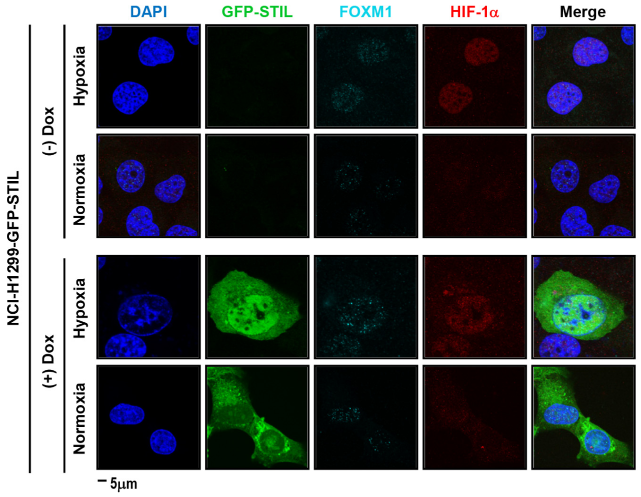

f

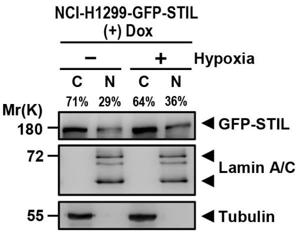

g

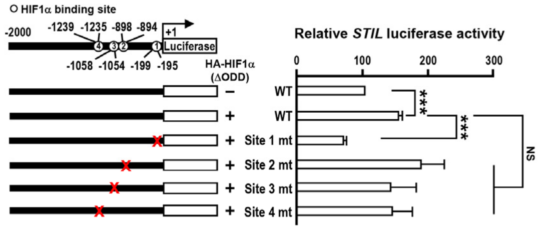

h

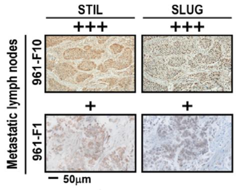

Concordant expression of STIL and SLUG

Type	n	Correlation	p-value
Metastatic lymph nodes	40	R=0.76	p< 0.0001

Supplementary Fig S8. The *STIL* DNA copy number and its DNA methylation status are not associated with lung cancers, and HIF1α could upregulate *STIL* expression under hypoxia. a The *STIL* DNA copy number was analyzed using an SNP array of 152 normal lung tissues, 86 lung cancer cell lines, and 1460 lung cancer specimens. The red lines indicated the median. The data of lung cancer cell lines were collected from EBI and the data of clinical specimens were collected from GEO datasets. **b** The DNA methylation status of *STIL* was measured by 450K methylation array involving 6 normal lung cell lines and 69 lung cancer cell lines, and 256 normal lung tissues and 124 lung cancer tissues; the data were derived from GEO datasets. The methylation status is represented by the β-value. **c** HIF1α protein levels were analyzed by Western blotting in CL1-0, CL1-3, and CL1-5 cells under normoxia. **d** STIL protein levels in cytoplasmic (C) and nuclear (N) fractions analyzed by Western blotting in CL1-0 cells under the normoxic (20% O_2) or hypoxic (1% O_2) condition. Lamin A/C and tubulin were used as nuclear and cytoplasmic markers, respectively. The percentage of subcellular distribution is shown. **e** The confocal images of NCI-H1299-based GFP-STIL-inducible cells were treated with or without Dox under hypoxic or normoxic conditions. **f** GFP-STIL protein levels in cytoplasmic (C) and nuclear (N) fractions analyzed by Western blotting in NCI-H1299 overexpressing GFP-STIL under Dox treatment for 48 hours. Lamin A/C and tubulin were used as nuclear and cytoplasmic markers, respectively. The percentage of subcellular distribution is also shown. **g** *STIL* promoter activity was measured by reporter assay in CL1-0 cells transiently transfected with pGL3-*STIL* promoter-driven luciferase constructs containing the wild-type HIF1α, the HIF1α DNA-binding site mutants (indicated by red X), and/or the HA-HIF1α (ΔODD) construct under normoxia. Data represent the mean ± SD (n = 3 independent experiments). Significance is determined by t-test (NS: not significant; ***$p < 0.001$). **h** Clinical correlation between STIL and SLUG expression in 40 metastatic lymph nodes, as analyzed by IHC and Pearson's correlation was included (R=0.76). Scale bar: 50 μm.
